# Freeze-Dried Immobilized Kefir Culture in Low Alcohol Winemaking

**DOI:** 10.3390/foods9020115

**Published:** 2020-01-21

**Authors:** Anastasios Nikolaou, Georgios Sgouros, Gregoria Mitropoulou, Valentini Santarmaki, Yiannis Kourkoutas

**Affiliations:** Laboratory of Applied Microbiology & Biotechnology, Department of Molecular Biology & Genetics, Democritus University of Thrace, GR-68100 Alexandroupolis, Greece; anikol@mbg.duth.gr (A.N.); gsgouros@mbg.duth.gr (G.S.); grigoriamitropoulou@gmail.com (G.M.); valentina.2@windowslive.com (V.S.)

**Keywords:** immobilization, freeze-drying, cryoprotectants, volatiles, next generation sequencing

## Abstract

Low alcohol wines represent a rising trend in the global market. Since for ethanol removal, certain physicochemical methods that negatively affect wine quality are applied, the aim of this present study was to evaluate the efficiency of freeze-dried, immobilized kefir culture on natural supports (apple pieces, grape skins and delignified cellulosic material) in low alcohol winemaking at various temperatures (5–30 °C). Initially, genetic analysis of kefir culture was performed by Next Generation Sequencing. There was an immobilization of kefir culture on grape skins-enhanced cell survival during freeze-drying in most cases, even when no cryoprotectant was used. Simultaneous alcoholic and malolactic fermentations were performed in repeated batch fermentations for >12 months, using freeze-dried free or immobilized cells produced with no cryoprotectant, suggesting the high operational stability of the systems. Values of great industrial interest for daily ethanol productivity and malic acid conversion [up to 39.5 g/(Ld) and 67.3%, respectively] were recorded. Principal Component Analysis (PCA) showed that freeze-drying rather than the fermentation temperature affected significantly minor volatiles. All low alcohol wines produced were accepted during the preliminary sensory evaluation.

## 1. Introduction

Low alcohol wines (alcohol content ≤ 10.5% vol) represent a new steadily rising trend in the global wine market driven by consumers’ social and economic interests [1]. Low alcohol wines can be classified into different categories based on their alcoholic strength, although the classification can vary greatly between countries. However, since low alcohol concentrations are difficult to achieve, wines are treated with physicochemical methods that negatively affect their quality [2].

Malolactic (ML) fermentation is known to occur naturally under normal conditions in wines, resulting in reduced acidity, microbial stability and improved sensory characteristics. However, it is a difficult process that may lead to delay or even failure. Thus, simultaneous alcoholic and malolactic fermentation that can be accomplished by yeasts in association with ML bacteria is usually suggested [3].

Kefir is a consortium of various yeasts, lactic acid and acetic acid bacteria [4,5,6] that co-exist symbiotically. Kefir culture has already been successfully used for cider fermentation [7], whereas recently it was assessed in simultaneous alcoholic and ML wine fermentations at various temperatures [8].

The use of wet cultures, on the other hand, is incompatible with modern industrial and commercial needs, while freeze-dried cultures are considered preferable due to significant technological advantages (longer preservation times, resistance to microbial contamination, easy-handling during storage, etc.). However, freeze-drying very often leads to a significant reduction of cell viability [9]. To overcome such deficiencies, cell immobilization is suggested, as it results in the maintenance of viability during the freeze-drying process [10], along with the operational stability of both wet and freeze-dried cells [10,11], and is also associated with multiple technological advantages (enhanced fermentation productivity, application of continuous configurations and cell recycling, improvement of product quality, etc.) [12]. Many food-grade, natural supports have been successfully tested for the immobilization of yeasts and ML bacteria in batch [13,14,15,16,17] or continuous configurations [18,19,20].

Recently, immobilized kefir culture on apple pieces, delignified cellulosic material (DCM) and grape skins, was assessed in simultaneous alcoholic and ML cider and wine fermentations [7,8]. However, these studies involved only wet immobilized kefir cultures, a technology hardly accepted by the industrial sector. Although the use of freeze-dried, immobilized cells has been investigated in winemaking [10,21] and brewing [22], leading to products with an improved taste and aroma, the optimization of the process (e.g., the use of cryoprotectants), and verification of the fermentation efficiency of newly prepared freeze-dried immobilized cultures, always constitute issues of great importance, due to viability and metabolic activity losses associated with the freeze-drying procedure [23,24].

In this vein, the aim of the present study was to evaluate the suitability of freeze-dried, immobilized kefir culture in the production of low alcohol wines. Data supporting the ability of the freeze-dried kefir culture to conduct simultaneous alcoholic and ML fermentations in low alcohol winemaking, and quality improvement, are presented.

## 2. Materials and Methods

### 2.1. Kefir Culture and Genetic Analysis

Kefir culture belongs to the microbial collection of the Laboratory of Applied Microbiology and Biotechnology of the Department of Molecular Biology and Genetics, Democritus University of Thrace (Alexandroupolis,, Greece), and was isolated by a traditional kefir drink originated by Caucasus, Armenia.

Genetic analysis of kefir culture was determined by next generation DNA sequencing as follows: Initially, DNA extraction was carried out as previously described [7]. Then, universal bacterial primers 27Fmod (5′ AGR GTT TGA TCM TGG CTCA G 3′) and 519Rmodbio (5′ GTN TTA CNG CGG CKG CTG 3′) were used to amplify the V1–V3 region of the 16S rRNA gene. Similarly, ITS1F (5′ CTT GGT CAT TTA GAG GAA GTA A 3′) and ITS4R (5′ TCC TCC GCT TAT TGA TAT GC 3′) were used for 18S and internal transcribed spacer (ITS) eukaryotic regions amplification. Polymerase chain reaction (PCR) was carried out using HotStarTaq Plus Master Mix Kit (Qiagen, Germantown, MD, USA): 94 °C for 3 min, followed by 30 cycles of 94 °C for 30 sec, 53 °C for 40 sec and 72 °C for 1 min, with a final elongation step at 72 °C for 5 min.

Library preparation was performed following Illumina TruSeq DNA protocol (MR DNA, Shallowater, TX, USA) and next generation sequencing using Illumina Miseq platform (MR DNA), according to the manufacturer’s guidelines. All sequence data derived were processed using MR DNA analysis pipeline (MR DNA) and final operational taxonomic units (OTUs) were taxonomically classified using BLASTn against a curated database derived from the Ribosomal Database Project (RDP-II) and the National Center for Biotechnology Information (NCBI).

### 2.2. Immobilization of Kefir Culture

Immobilization of kefir culture on natural supports [apple pieces, delignified cellulosic material (DCM), grape skins] was performed as recently described [8].

### 2.3. Freeze-Drying

In order to investigate the effect of various cryoprotectants on cell viability during freeze-drying, sugar solutions (glucose, fructose, sucrose, lactose and trehalose at concentrations of 10% and 25% *w*/*v*), glycerol solutions (10%, 25% and 50% *v*/*v*) and grape must (~10 °Be) were tested as previously described with few modifications [10,25]. All solutions were sterilized at 121 °C for 15 min prior to use.

Immobilized cells were transferred in sterile beakers and covered with the cryoprotective solutions for 1 h at room temperature. Then the solution was decanted and the immobilized cells were frozen overnight at −80 °C. Free cells were resuspended in each cryoprotective solution at a ratio of 1 mL/1 g of cells, and left at room temperature for 15 min before overnight freezing at −80 °C. Free and immobilized kefir culture with no cryoprotectant served as controls.

Frozen free kefir samples and immobilized cells on DCM and grape skins were freeze-dried on a BenchTop Pro (Virtis, SP Scientific, Warminster, PA, USA) for 24 h at ~30–35 Pa with the condenser temperature fixed at −101 °C. Immobilized cells on apple pieces were freeze-dried for 72 h due to the porous nature of the support.

### 2.4. Determination of Cell Viability after Freeze-Drying

Freeze-dried cells were rehydrated with sterilized water [25] and yeasts/molds, lactobacilli and lactococci counts were determined as described previously [7]. The % survival rate was calculated as logcfu/g after freeze-drying, divided by logcfu/g before freeze-drying, and multiplied by 100 as previously described [10].

### 2.5. Fermentations

Concentrated grape must of Roditis and Savatiano grape varieties was provided by the wine companies “B.G. Spiliopoulos S.A.” and “Georga’s Family”, respectively. Both musts were diluted to a final ~10 ± 0.5 °Be density (~170 ± 8.5 g/L sugars, 3.2 ± 0.2 g/L malic acid, total acidity 5.0 ± 0.5 g tartaric acid/L), mixed in a 1:1 ratio, and sterilized at 121 °C for 15 min prior to use.

After rehydration of the freeze-dried cultures, batch fermentations (250 mL) of grape must were carried out in 0.5 and 1 L batch bioreactors, using either freeze-dried free (10 g/L) or freeze-dried immobilized cells on natural supports (1420 g/L of apple pieces, 480 g/L of DCM and 500 g/L of grape skins), as previously described [8].

Repeated batch fermentations of grape must (250 mL) were carried out using freeze-dried cells with no cryoprotectants, as previously described [8]. In brief, three repeated batches at 30 °C, five at 20 °C and three at 5 °C were performed using freeze-dried free or immobilized cells in batch bioreactors. Repeated batch fermentations using wet cells were carried out as a control. Grape must was used to wash the cells, before the next batch fermentation.

All fermentations were carried out until all sugar content was utilized, or when no fermentation activity was observed (stuck fermentations).

### 2.6. Chemical Analyses

#### 2.6.1. Water Activity (a_w_), pH, Total and Volatile Acidity

Water activity (a_w_) of the freeze-dried samples was determined using the HygroLab 3 (Rotronic AG, Bassersdorf, Switzerland), according to the manufacturer’s guides.

The pH, total acidity and volatile acidity values were measured as previously described [7].

#### 2.6.2. Residual Sugars, Ethanol, Glycerol and Organic Acids

High-performance liquid chromatography (HPLC) was used to determine residual sugars, ethanol, glycerol, malic, lactic, acetic, citric and propionic acid concentration [7]. All fermentation parameters were calculated as recently described [7].

#### 2.6.3. Volatile by-Products

Major volatile by-products [acetaldehyde, ethyl acetate, 1-propanol, 2-methyl-1-propanol (isobutanol), 1-hexanol, 2-methyl-1-butanol (amyl alcohol), 3-methyl-1-butanol (isoamyl alcohol) and methanol] content was determined by gas chromatography, as previously described [7].

Minor volatile by-products were determined by headspace solid-phase microextraction (HS-SPME) gas chromatography–mass spectrometry (GC-MS) analysis, as recently described [8] using a GC/MS (6890N GC, 5973NetworkedMS MSD, Αgilent Technologies, Santa Clara, CA, USA) equipped with an HP-5MS column (30 m, 0.25 mm i.d., 0.25 μm film thickness).

### 2.7. Preliminary Sensory Evaluation

Low alcohol wine products were evaluated for their sensory attributes, and compared with a commercially similar variety product, as previously reported [8].

### 2.8. Statistical Analysis

Data regarding the cell viability and batch fermentations of freeze-dried cells were analyzed for statistical significance by 2-way analysis of variance (ANOVA) [the cryoprotectant used and the nature of kefir culture (free or immobilized) were considered as factors]. All other data were analyzed for statistical significance by 3-way analysis of variance [the state of the cells (wet or freeze-dried), the nature of kefir culture and the fermentation temperature, were considered as factors]. Duncan’s multiple range test was used to determine differences among results. Statistica v.10.0 (Stat Soft Inc., Tulsa, OK, USA) was used to generate significance at *p* < 0.05, coefficients and ANOVA Tables.

XLSTAT 2015.1 was used to compute the principal component analysis (PCA) algorithm [7].

## 3. Results and Discussion

### 3.1. Genetic Analysis of Kefir Culture by Next Generation DNA Sequencing

After being grown on synthetic medium, genetic analysis of kefir culture was carried out applying next generation DNA sequencing (NGS) (data not shown). 20 k sequence reads were performed using Illumina Miseq platform (MR DNA) for the 16S rRNA bacterial and 18S-ITS eukaryotic regions, respectively.

Bacterial sequence reads revealed four different phyla, highly representative of kefir culture [26,27]. Firmicutes [which include lactic acid bacteria (LAB)] were the predominant phylum, accounting for > 98% of the total sequences, Bacteriodetes comprised for 1%, while both Proteobacteria and Actinobacteria phyla accounted for < 1% [5]. At family level, 96% of the sequence reads accounted for *Lactobacillaceae* [26], 1% for *Clostridiaceae* and the rest reads were shared between *Enterobacteriaceae*, *Bacteroidaceae*, *Acetobacteraceae*, *Enterococcaceae*, *Streptococcaceae*, *Bifidobacteriaceae*, *Propionibacteriaceae*, *Pseudomonadaceae*, *Rikenellaceae*, *Lachnospiraceae* and *Ruminococcaceae* in very low percentages (<1% for each) [27]. Regarding genus level, 96% of the reads belonged to *Lactobacillus* [28], while other genera that are not usually associated with kefir culture (*Allistipes*, *Allobaculum*, *Enterococcus*, and *Pseudomonas*) were detected in very low levels (<1%), and could indicate a possible environmental contamination [26]. Similarly, *Lactobacillus kefiri* (95%) was the dominant species detected [29,30], while other bacteria belonging to *Lactobacillus* and *Lactococcus* genera accounted for a very small portion (<1%) [6].

On the other hand, eukaryotic sequence reads showed lower diversity, as >99.5% was associated with the Ascomycota phylum, and linked to the *Saccharomycetaceae* family of yeasts (>99.5%) [26]. Predominant genera that exist in kefir culture, like *Kluyveromyces* (97%) and *Saccharomyces* (2%) were detected [31,32,33], whereas the rest of the reads (<1% in total) were split between other genera (the fungi *Kazachstania* and *Torulaspora*). A few genera that do not usually constitute members of kefir culture (*Eurotium*, *Malassezia*, *Cryptococcus* and *Zygosaccharomyces*) [34] were also identified. Species of *Kluyveromyces lactis* (97%) and *Saccharomyces cerevisiae* (2%) assembled the majority of the sequence reads. Other characteristic kefir species (*Hanseniaspora guilliermondii* and *Torulaspora delbrueckii*) were found in very low levels (<1%) [5,35].

Many species associated to probiotic or beneficial properties have been previously isolated from kefir culture [36,37], ascertaining the safety of the wines. Specifically, *Lactobacillus kefiri* strains have been associated with protective effects against *Salmonella*, *Escherichia coli* (*E. coli*) and other pathogenic infections and inhibitory action against *C. difficile* toxins [38]. Similarly, *Saccharomyces* and *Kluyveromyces* species are the most extensively studied kefir yeasts, and are known for their antioxidant properties [39]. Moreover, the fermentation of plant-based foods has been shown to improve antioxidative activity in the final product [40], while solids of grape origin could act as prebiotics and potentiate the antioxidant activity of kefir-derived yeasts [39].

Overall, ethanol, microbial metabolites and other by-products (organic acids, carbon dioxide, peroxides, aroma compounds, acetaldehyde, antibiotic substances, bacteriocins, etc.) are produced during kefir-induced fermentation. These compounds may act independently or synergically to confer various health benefits [41], inhibit pathogenic microbes [42,43], enhance shelf life by securing microbial safety and contribute to the taste, aroma, texture and the nutritional attributes of the fermented products [44].

### 3.2. Freeze-Drying and Cell Viability Determination

**Figure 1:**
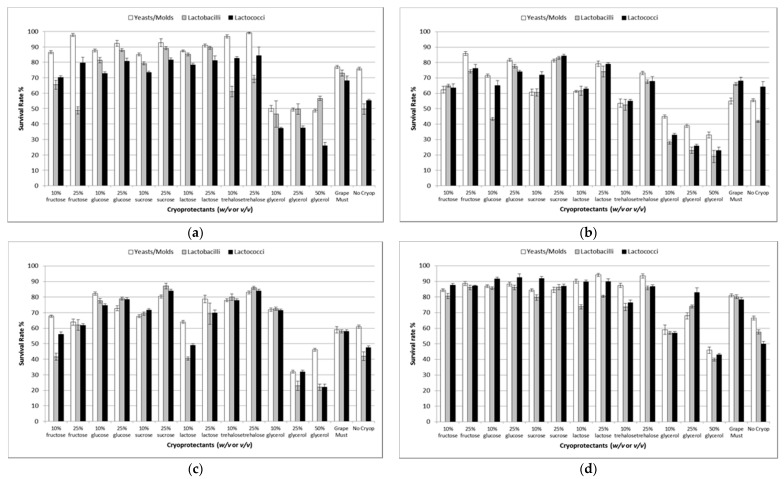
Effect of various cryoprotectants on the survival rate (%) of freeze-dried free and immobilized kefir cells. (**a**) Free kefir culture, (**b**) Immobilized kefir cells on apple pieces, (**c**) Immobilized kefir cells on delignified cellulosic material (DCM), (**d**) Immobilized kefir cells on grape skins. Freeze-dried kefir cells without any cryoprotectant were used as control samples.

Initially, kefir culture was immobilized on natural supports, and then the effect of various cryoprotectants during freeze-drying on cell survival was investigated. As the ability of kefir culture to perform both alcoholic and ML fermentation was of interest, it was important to achieve the high survival rates of both yeasts and ML bacteria. Thus, the optimum conditions of a nonselective treatment, such as freeze-drying, were studied. The results are presented in Figure 1. Both the cryoprotectants and the nature of kefir culture (free or immobilized cells) had a significant (*p* < 0.05) effect on cell survival, and a strong (*p* < 0.05) interaction between the two factors was also observed. Cell immobilization had a negative effect upon the yeast survival rate in most cases (especially cell immobilization on apple pieces [9]), probably due to the longer time required for drying (72 h for immobilized cells on apple pieces in contrast to 24 h in free and immobilized cells on DCM and grape skins), owing to the hydrophilic nature of the immobilization support or because of yeast cells’ size and structure [45]. Only cell immobilization on grape skins had a significant (*p* < 0.05) positive effect on the survival of yeasts/molds when 25% *w*/*v* lactose, 10% and 25% *v*/*v* glycerol and grape must were used as cryoprotectants. Immobilization on apple pieces resulted in higher (*p* < 0.05) survival rates of lactobacilli when 25% *w*/*v* fructose solution was used as a cryoprotectant. Similarly, cell immobilization on DCM had a significant (*p* < 0.05) positive effect on the survival of lactobacilli when 25% *w*/*v* fructose, 10% and 25% *w*/*v* trehalose, and 10% *v*/*v* glycerol solutions, were used as cryoprotectants, and on the survival of lactococci when 10% *v*/*v* glycerol was applied [10,46]. Likewise, immobilization on grape skins also had a significant (*p* < 0.05) positive effect upon the survival of lactobacilli when sugar (fructose, trehalose), glycerol solutions (regardless the concentration used) and grape must were tested [47]. Remarkably, even when no cryoprotectant was applied in freeze-drying, immobilization on grape skins enhanced significantly (*p* < 0.05) the viability of lactobacilli. In the same manner, immobilization on grape skins and the use of cryoprotectants affected (*p* < 0.05) lactococci viability in most cases, with the exception of trehalose (10 and 25% *w*/*v*), and when no cryoprotective solution was used.

Water activity (a_w_) ranged in very low values (0.03–0.28) in all cases [9,25]. Although water removal may cause a decrease in survival rate, cell immobilization seemed to protect cells during freeze-drying [48], or even enhance cell survival in certain cases. Noticeably, survival rates ranged in similar or higher levels to previously published results concerning the freeze-drying of free kefir cells [23]. However, different cryoprotectants were used, and no data about different microbial genera were presented.

### 3.3. Fermentations

#### 3.3.1. Batch Fermentations

The fermentation ability of freeze-dried kefir cells was tested in batch fermentations at 30 °C. Initial concentration of freeze-dried cells corresponding to equal cell numbers of wet cultures (prior freeze-drying) was applied, based on previous studies of our group [8], to allow comparison of the results. The fermentation kinetic data and organic acids profile are shown in Table 1. Fermentation time, ethanol concentration, ethanol productivity and yield, residual sugars and sugar conversion, glycerol and malic acid content, malic acid conversion, volatile acidity, and pH values were significantly (*p* < 0.05) affected by the nature of kefir culture (free or immobilized), and the cryoprotectant used, while a strong interaction (*p* < 0.05) was observed between the factors. Citric acid content and total acidity was only affected (*p* < 0.05) by the nature of kefir culture. Likewise, lactic acid concentration was only affected (*p* < 0.05) by the cryoprotectant used, although a strong interaction (*p* < 0.05) was noted between the two factors.

Immobilization on apple pieces and DCM led to shorter fermentation times compared to immobilized cells on grape skins and free cells in most cases, in accordance to previous results [49]. The highest fermentation time (*p* < 0.05) was observed in fermentations with freeze-dried free cells produced with no cryoprotectant, probably due to the potential damage of cells during freeze-drying, or to the lack of a protected microenvironment offered by immobilization [10,46,50]. Ethanol concentration values ranged up to 10.4% ± 0.1% vol and were lower in wines fermented by freeze-dried immobilized kefir cells on apple pieces, although not significantly in all cases. Εthanol productivity values up to 21.3 ± 0.4 g/(Ld) were recorded (in the case of freeze-dried, immobilized cells on DCM when 25% *w*/*v* sucrose was used as the cryoprotectant), which are acceptable by the wine industry [10,51].

The use of glycerol solutions as cryoprotectants led to significantly higher glycerol concentrations, especially when 25% and 50% *v*/*v* concentrations were used for the freeze-drying of immobilized cells on apple pieces (*p* < 0.05), probably due to the porous nature of the support. Thus, glycerol residue concentrations up to 47.4 ± 16.8 g/L were detected in the final products, affecting the quality negatively [22]. In the rest cases, glycerol concentration was significantly (*p* < 0.05) lower. Total and volatile acidity ranged in usual levels for wines in all new products.

Μalic acid conversion rates similar to other studies were recorded [15,52] and ranged in levels accepted by the industrial sector [53]. The highest values (60.9 ± 12.7%) were observed at fermentations with freeze-dried immobilized cells on apple pieces when 10% *v*/*v* glycerol was used as the cryoprotectant. Lactic acid content was detected in very low concentrations (≤1.0 g/L) in all cases. Acetic acid, on the contrary, was detected only in fermentations with no cryoprotectant applied.

#### 3.3.2. Repeated Batch Fermentations

The suitability of freeze-dried kefir culture was further tested in simultaneous alcoholic and ML repeated batch fermentations for low alcohol wine production and compared to wet cells. Wine fermentations at high temperatures (30 °C) lead to drastic operational cost reduction in tropical countries, or during the summer periods of many non-tropical regions [8], while fermentations at low temperatures (<20 °C) contribute positively in wine quality. Moreover, the use of expensive cryoprotectants in industrial practice is considered ambiguous [20], while their absence may result in further operational cost reduction [25]. Thus, repeated batch fermentations at various temperatures (5–30 °C) were performed using freeze-dried kefir cells produced with no cryoprotectants, due to the great economic and technological interest. Repeated batch fermentations using wet kefir cells served as control.

Although winemaking using wet immobilized kefir culture on apple pieces, DCM and grape skins, was recently proposed [8], this is the first report on low alcohol wine production using freeze-dried kefir cells. Fermentation kinetic data and important enological parameters are presented in Table 2 and Table 3.

Fermentation time, ethanol productivity, the content of lactic, acetic and propionic acid, glycerol concentration, total and volatile acidity, and pH were affected significantly (*p* < 0.05) by the state of the cells (wet or freeze-dried), the nature of kefir cells (free or immobilized) and the fermentation temperature, while strong interactions (*p* < 0.05) were observed between the factors. On the other hand, ethanol concentration was affected (*p* < 0.05) by the culture’s nature and the fermentation temperature. Ethanol yield, malic acid concentration and malic acid conversion were affected (*p* < 0.05) only by the fermentation temperature. Likewise, residual sugars and sugar conversion were affected significantly (*p* < 0.05) by the nature οf the culture, and the fermentation temperature and citric acid concentration was affected (*p* < 0.05) by the state of the cells and the nature of the culture. Nevertheless, strong interactions (*p* < 0.05) were noted between the two factors in all cases.

The fermentation efficiency of freeze-dried free or immobilized cells was tested in 11 repeated batch fermentations for a period greater than 12 months. Low alcohol wine production at 5 °C using either wet or freeze-dried cells resulted in significantly (*p* < 0.05) higher fermentation times compared to other temperatures, as expected [8,11,14,16,17,46,54,55], but in a range usually observed in industrial applications [56,57]. Thus, low (*p* < 0.05) ethanol productivity was noted at 5 °C, while it was significantly (*p* < 0.05) higher at 20 and 30 °C [up to 39.5 g/(Ld)], ranging in values similar or greater than usually noticed in wine fermentations [8,10,16], but in most cases several fold higher than observed in traditional practice [20,58]. Ethanol content ranged 4.5%–10.5% (*v*/*v*) depending on fermentation temperature, and the highest values for freeze-dried cells were observed when immobilized kefir cells on grape skins were used [13,59], although not significantly in all cases. Notably, freeze-dried kefir cells resulted in improved fermentation kinetic data [10,23,25,48] and enhanced operational stability in low alcohol winemaking, as repeated batch fermentations proceeded, despite the changes in fermentation temperature [21,51,58]. The above results are of great interest for the industrial sector, since they indicate efficient wine production [60].

Glycerol concentration ranged in usual levels for wines, although in most cases values >5.2 g/L were determined, contributing to the wine “sweetness” [61]. Total acidity also ranged in the usual levels for wines, while volatile acidity was found within the legal limits (<1.0 g acetic acid/L) in most cases [62]. In all wines produced at 5 °C [8], as well as in wines produced by wet immobilized cells on DCM at 30 °C, volatile acidity was increased compared to other temperatures, although not significantly in all cases. Nevertheless, volatile acidity never exceeded 2.1 g acetic acid/L, a level often noticed in special wine flavor profiles, like Canadian “ice wines” [61,63].

Malic acid conversion up to 67.3% was recorded, whereas the malolactic activity of all kefir cells was maintained during the whole duration of the fermentations [7,8,60]. Significantly higher (*p* < 0.05) values were observed at fermentations with freeze-dried, immobilized cells on grape skins at 5 °C (Table 3). Of note, similar malic degradation values were previously reported [7,8,60,64]. A significantly higher (*p* < 0.05) content of lactic acid (4.1 g/L) was noted in fermentations with wet, immobilized cells on DCM at 30 °C (at the first batch only), but it was significantly (*p* < 0.05) reduced in subsequent fermentations, as previously shown [7]. However, high concentrations of lactic acid are not unusual in winemaking after ML fermentation [8,60,65,66]. Acetic acid is known to contribute to the high volatile acidity [56]. It was detected in levels up to 2.2 g/L in wines produced by freeze-dried free and immobilized cells on apple pieces at 5 °C, while similar values have been recently observed in wine fermentations with wet free or immobilized kefir cells [8]. However, in most cases, it was present at significantly lower (*p* < 0.05) levels. High acetic acid concentration (up to 2.1 g/L) is usually detected in special type wines [61], although it is suggested not to exceed 0.3 g/L, in order to contribute to the taste and odor complexity [56].

### 3.4. Volatiles

The development of a unique, aromatic profile is always the desirable aim for the wine industry. Hence, major and minor volatile by-products analysis was performed.

#### 3.4.1. Major Volatiles

All major volatiles detected are presented in Table 4. Acetaldehyde, amyl and isoamyl alcohol, 1-propanol, isobutanol, 1-hexanol and methanol content were affected significantly (*p* < 0.05) by the three factors, and strong interactions (*p* < 0.05) were observed. On the contrary, ethyl acetate concentration was affected (*p* < 0.05) by the state of the cells, the nature of kefir culture and their interaction.

Acetaldehyde was detected at low levels (≤88 mg/L) in all products [20], and ethyl acetate content remained at < 50 mg/L, contributing pleasantly to its fragrance complexity [56]. Ethyl acetate concentration was increased in wines produced by freeze-dried, immobilized cells on DCM compared to wines produced by immobilized cells on other supports (both wet and freeze-dried), while the significantly highest (*p* < 0.05) concentration was detected in wines produced by freeze-dried, immobilized cells on DCM at 30 °C, in agreement to previous studies [22,51].

Higher alcohols (amyl and isoamyl alcohols, 1-propanol, isobutanol) contribute to the aromatic complexity at low concentrations, but higher concentrations may cover the aromatic profile of the wine [67]. Their content was found in the usual levels in wines produced by wet kefir cells, and the reduction noted along with the temperature decrease is well documented and associated with an improvement in the wine quality [8,11,14]. Increased (*p* < 0.05) content of higher alcohols was detected in wines produced by freeze-dried, immobilized cells on grape skins at 30 °C, compared to other freeze-dried cells [8], as well as at 20 and 5 °C, although not significantly in all cases. However, they were still present at levels usual for wines [68], and were significantly reduced (*p* < 0.05) at 5 °C in most cases [7,11,16], with the exception of 1-propanol. Winemaking practices (high fermentation temperatures, oxygen presence, skins and solids in the fermenting juice) can influence the formation of higher alcohols during fermentation [56], even though their production might be enhanced at cooler temperatures [56]. 1-hexanol and methanol were present in extremely low levels in all cases (<50 mg/L). Methanol is known for its toxicity [56], and ranged in very low levels in all cases. Similar results were previously reported in wines produced by wet immobilized kefir cells on natural supports [8], which is considered a positive factor.

#### 3.4.2. Minor Volatiles

All low alcohol products were evaluated regarding their aromatic profile by headspace solid-phase microextraction gas chromatography–mass spectrometry (HS-SPME/GC-MS) analysis. Semi-quantitative results of the total volatile compounds detected are presented in Table 5.

In total, 52 compounds (Table S1) were identified, mostly esters, organic acids, alcohols, and carbonyl compounds. The nature of kefir culture and the fermentation temperature affected significantly (*p* < 0.05) ester, carbonyl compound, and total volatiles content. On the other hand, the state of the cells and the nature of kefir culture had a significant (*p* < 0.05) effect on organic acids and miscellaneous compounds detected. Alcohols detected were significantly (*p* < 0.05) affected by the three factors. Also, strong interactions (*p* < 0.05) between factors were noted for acids and carbonyl compounds.

Esters concentration (known for its positive contribution to wine aroma) was increased at 20 and 5 °C compared to higher fermentation temperatures [68]. Mostly acetates of higher alcohols were identified, known for their fruity attributes [69], as well as ethyl esters of fatty acids, known to add wax and honey notes [70] (Table S1), which have a positive impact on the final product [68]. Ethyl propanoate (blackberry notes), isoamyl acetate (banana-like scent), isobutyl acetate (fresh and fruity character), ethyl butyrate (apple-peel attributes), 3-methylbutyl acetate (banana attributes), ethyl hexanoate (pineapple notes), ethyl octanoate and ethyl decanoate (floral, fruity, musty notes) and 2-phenylethyl acetate (banana-apple aroma) [56,71] were detected in all samples, and their synthesis is favored at low fermentation temperatures [67]. Likewise, 2-methylbutyl acetate (peer flavors), ethyl dodecanoate (dried fruit, smokey, earthy, toasty aroma), ethyl hexadecanoate (candy, herbal, spicy aroma), and ethyl-9-decenoate (pleasant odor) [68,71] were identified in most cases, and are also present in wines [8,10].

As fatty acids have low odor threshold values, this might have a flavor impact in wine. An increase (*p* < 0.05) in the content of organic acids, previously associated with an improvement of wine quality [68], was noted in low alcohol wines produced by wet cells immobilized on DCM and in wines produced by freeze-dried cells immobilized on grape skins, although not significant in all cases. Hexanoic acid, known for its positive impact [10], octanoic acid (providing rancid, butter, floral, cabbage aroma) and *n*-decanoic acid contributing to rancid, phenolic notes [71] were identified (Table S1).

Only few carbonyl compounds were detected (Table S1). Benzaldehyde (bitter almond odor) was mostly present in wines produced at higher fermentation temperatures, and *β*-damascenone, with the complex smell of flowers, tropical fruit and stewed apple, was detected at low concentrations [70,72]. Nonanal with a fruity or floral odor that might have a positive effect on the product quality was also detected in a few samples [73].

Regarding alcohols (Table S1), 2,3-butanediol with a bittersweet taste [69] was identified mainly in products produced by wet cells, but is unlikely to be of important sensory significance in wine [56]. 2-phenyl-ethanol with a characteristic roses’ aroma, on the other hand, was found in all wines. Citronellol (floral, fruity and citrus notes) [74,75] was detected in some cases at levels higher than its perception threshold (0.018 mg/L) [70], while nerolidol, providing hay flavors, was identified in several samples in low quantities [56,69].

Concerning the miscellaneous compounds (Table S1), 1,1-diethoxy-ethane (refreshing, fruit and green odor) is the only acetal that might contribute to the wine aroma, as acetals have a minor impact upon the wine bouquet [56,68]. On the contrary, hydrocarbons detected are considered mostly insignificant to the wine aroma [68].

#### 3.4.3. Chemometrics

**Figure 2:**
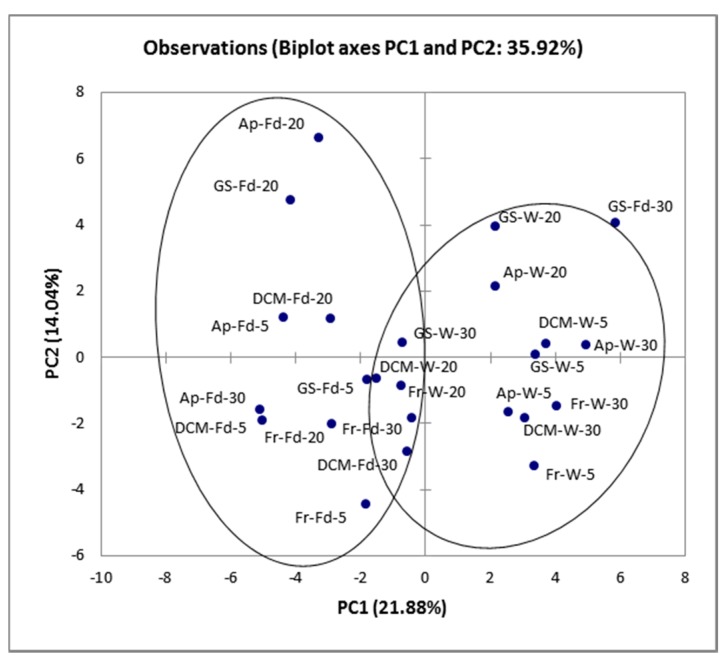
Principal component analysis (PCA) plot of minor volatiles isolated by low alcohol wines fermented by wet or freeze-dried free or immobilized kefir culture. Fr: low alcohol wine fermented by free kefir culture, Ap: low alcohol wine fermented by immobilized kefir culture on apple pieces, DCM: low alcohol wine fermented by immobilized kefir culture on delignified cellulosic material (DCM), GS: low alcohol wine fermented by immobilized kefir culture on grape skins. The state of the cells is referred as W: Wet cells or Fd: Freeze-dried cells, after the sample’s name. The fermentation temperature is indicated at the end of the sample code.

The principal component analysis (PCA) algorithm applied to the HS-SPME GC/MS results showed that the state of the cells rather than the fermentation temperature affected significantly volatile composition, since two distinct groups (wines fermented with wet or freeze-fried cells) were observed (Figure 2). Likewise, fermentations performed at 30 °C are presented at the right side of the graph, while fermentations performed at 20 and 5 °C were mostly gathered towards the left and the bottom side of the graph.

### 3.5. Preliminary Sensory Evaluation

All wines were evaluated for their quality attributes and compared to a commercially available product (Roditis-Savatiano Varietal Dry White Wine, Cellar S.A., Athens, Greece). According to the results (Table 6), the state of the cells, the nature of kefir culture and the fermentation temperature affected significantly (*p* < 0.05) the aroma of all low alcohol wines produced, while strong interactions (*p* < 0.05) were observed between all factors. On the other hand, the taste and overall quality were affected significantly (*p* < 0.05) by the nature of kefir culture, the fermentation temperature and their interaction, but a strong interaction (*p* < 0.05) between all three factors was recorded.

Despite the absence of post-fermentation treatments, all products exhibited high clarity, and were accepted by the tasters when compared to the commercial product. The new wines were characterized by a fruity and wine-like aroma, while in some cases, products fermented with immobilized cells on DCM and grape skins presented a piquant and a spirituous aroma (data not shown), respectively. A sour or sweet/sour taste was mostly considered, whereas in products produced at 5 °C, some bitter notes were detected (data not shown). Notably, no vinegar taint was detected in wines produced at 5 °C, regardless the relatively high volatile acidity and high content of acetic acid, as previously stated [8]. Most products were light-bodied with a mild aftertaste, except for wines produced by immobilized cells on DCM and grape skins which were medium-bodied with stronger full aftertaste (data not shown).

Remarkably, the highest overall quality ranking (similar to or even higher than the available commercial product) was attributed to low alcohol wines produced at 30 °C by immobilized cells (wet or freeze-dried) on DCM and grape skins.

## 4. Conclusions

Freeze-dried, immobilized kefir culture proved suitable for conducting alcoholic and ML low alcohol wine fermentations simultaneously, at various temperatures. The genetic analysis of kefir culture by next generation DNA sequencing showed that the predominant species identified were members of *Kluyveromyces lactis*, *Saccharomyces cerevisiae* and *Lactobacillus kefiri*. Immobilization on grape skins enhanced the cell survival in most cases and most importantly of lactobacilli, when no cryoprotectant was used. Although the fermentation ability of freeze-dried cells was successfully tested in fermentations at 30 °C, the use of cryoprotectants was rejected due to residues detected in the final products. Repeated batch fermentations using freeze-dried kefir cells produced with no cryoprotectant at a wide temperature range suggested the high operational stability of the systems. The state of the cells (wet or freeze-dried) rather than the fermentation temperature affected significantly minor volatiles. Nevertheless, more research data are required in order to meet modern industrial and commercial needs, especially in issues associated with the storage and maintenance of cell viability in periods between winemaking seasons.

## Figures and Tables

**Table 1:** Fermentation parameters and organic acids profile of low alcohol wines produced using freeze-dried free or immobilized kefir culture at 30 °C.

Nature of Kefir Culture	Cryoprotectants (*w*/*v* or *v*/*v*)	Fermentation Time (h)	Ethanol Concentration (% vol)	Glycerol (g/L)	Residual Sugars (g/L)	Ethanol Productivity [g/(Ld)]	Ethanol Production Yield	Conversion (%)	Malic Acid † (g/L)	Lactic Acid (g/L)	Malic Acid Conversion (%)	Acetic Acid (g/L)	Citric Acid (g/L)	Total Acidity (g Tartaric/L)	Volatile Acidity (g Acetic/L)	pH
Free cells	10% fructose	110 ± 20	9.8 ± 0.8	5.1 ± 1.4	1.6 ± 0.3	17.1 ± 1.7	0.45 ± 0.02	99.1 ± 0.2	2.7 ± 0.3	0.5 ± 0.1	17.0 ± 5.8	Nd	1.4 ± 0.7	4.1 ± 0.6	0.33 ± 0.04	3.4 ± 0.1
	25% fructose	140 ± 26	9.2 ± 1.3	5.3 ± 1.4	8.0 ± 1.6	12.5 ± 0.5	0.44 ± 0.04	95.4 ± 1.1	2.1 ± 0.4	0.6 ± 0.1	37.6 ± 10.6	Nd	1.6 ± 0.8	3.8 ± 0.6	0.30 ± 0.04	3.5 ± 0.1
	10% glucose	110 ± 19	9.7 ± 1.0	4.8 ± 1.3	0.9 ± 0.2	16.9 ± 1.2	0.44 ± 0.03	99.5 ± 0.1	2.4 ± 0.4	0.3 ± 0.1	26.2 ± 9.4	Nd	1.3 ± 0.6	3.9 ± 0.6	0.39 ± 0.05	3.4 ± 0.1
	25% glucose	140 ± 24	9.2 ± 1.0	6.3 ± 1.7	11.2 ± 2.2	12.6 ± 0.7	0.45 ± 0.03	93.6 ± 1.5	1.9 ± 0.5	0.6 ± 0.1	44.0 ± 11.9	Nd	1.5 ± 0.7	3.5 ± 0.5	0.60 ± 0.08	3.6 ± 0.1
	10% sucrose	110 ± 20	9.3 ± 1.5	4.0 ± 1.1	0.8 ± 0.2	16.1 ± 0.5	0.42 ± 0.05	99.5 ± 0.1	2.2 ± 0.4	0.2 ± 0.1	34.4 ± 9.3	Nd	0.9 ± 0.4	4.2 ± 0.7	0.39 ± 0.05	3.4 ± 0.1
	25% sucrose	132 ± 23	9.3 ± 1.1	5.7 ± 1.5	8.4 ± 1.7	13.5 ± 0.9	0.45 ± 0.03	95.1 ± 1.1	2.3 ± 0.4	0.6 ± 0.1	30.6 ± 8.8	Nd	1.8 ± 0.9	3.8 ± 0.6	0.42 ± 0.05	3.5 ± 0.1
	10% lactose	96 ± 17	10.0 ± 0.6	5.4 ± 1.5	1.5 ± 0.3	20.0 ± 2.4	0.46 ± 0.01	99.2 ± 0.2	2.5 ± 0.4	0.2 ± 0.1	24.4 ± 8.8	Nd	1.2 ± 0.6	4.5 ± 0.7	0.39 ± 0.05	3.4 ± 0.1
	25% lactose	96 ± 18	10.1 ± 0.4	5.5 ± 1.5	1.5 ± 0.3	20.2 ± 2.9	0.46 ± 0.01	99.1 ± 0.2	2.7 ± 0.4	0.4 ± 0.1	18.0 ± 8.1	Nd	1.1 ± 0.5	4.5 ± 0.7	0.51 ± 0.06	3.3 ± 0.2
	10% trehalose	140 ± 26	9.2 ± 1.2	5.6 ± 1.5	7.9 ± 1.6	12.6 ± 0.7	0.44 ± 0.04	95.5 ± 1.0	2.2 ± 0.5	0.6 ± 0.1	35.0 ± 12.0	Nd	1.7 ± 0.7	3.8 ± 0.6	0.48 ± 0.06	3.5 ± 0.1
	25% trehalose	120 ± 21	8.2 ± 2.1	5.8 ± 1.5	13.1 ± 3.3	12.9 ± 1.0	0.40 ± 0.08	92.4 ± 2.2	2.2 ± 0.4	0.6 ± 0.1	33.8 ± 10.3	Nd	1.4 ± 0.6	3.9 ± 0.6	0.45 ± 0.06	3.4 ± 0.1
	10% glycerol	144 ± 24	7.3 ± 1.9	2.9 ± 0.8	1.1 ± 0.2	9.6 ± 0.8	0.34 ± 0.09	99.4 ± 0.1	2.0 ± 0.4	0.3 ± 0.1	38.2 ± 14.0	Nd	1.0 ± 0.5	4.2 ± 0.7	0.36 ± 0.05	3.2 ± 0.1
	25% glycerol	156 ± 29	10.0 ± 0.6	4.5 ± 1.2	1.2 ± 0.2	12.3 ± 1.6	0.46 ± 0.01	99.3 ± 0.2	2.9 ± 0.3	0.6 ± 0.1	11.9 ± 5.6	Nd	1.1 ± 0.5	4.7 ± 0.7	0.33 ± 0.04	3.3 ± 0.1
	50% glycerol	140 ± 28	10.1 ± 0.6	4.6 ± 1.2	1.1 ± 0.2	13.9 ± 2.0	0.46 ± 0.01	99.4 ± 0.1	2.6 ± 0.4	0.3 ± 0.1	21.0 ± 8.9	Nd	1.3 ± 0.6	5.0 ± 0.8	0.36 ± 0.05	3.3 ± 0.1
	Grape Must	120 ± 22	9.6 ± 1.2	4.4 ± 1.2	0.8 ± 0.2	15.3 ± 0.9	0.44 ± 0.04	99.6 ± 0.1	2.3 ± 0.5	0.2 ± 0.1	30.1 ± 12.9	Nd	1.0 ± 0.5	4.4 ± 0.7	0.42 ± 0.05	3.4 ± 0.1
	No Cryoprotectant	240 ± 44	8.8 ± 2.0	6.7 ± 1.8	5.5 ± 1.1	6.9 ± 0.3	0.41 ± 0.08	96.8 ± 0.7	2.5 ± 0.5	1.0 ± 0.2	25.7 ± 11.6	1.0 ± 0.2	0.8 ± 0.4	4.4 ± 0.7	0.90 ± 0.11	3.7 ± 0.1
Immob. cells on apple pieces	10% fructose	96 ± 18	7.2 ± 1.8	4.8 ± 1.7	1.4 ± 0.7	14.1 ± 1.0	0.34 ± 0.08	99.2 ± 0.4	2.8 ± 0.2	0.5 ± 0.2	13.9 ± 6.1	Nd	1.0 ± 0.1	3.6 ± 0.8	0.21 ± 0.04	3.3 ± 0.1
	25% fructose	75 ± 13	6.9 ± 1.8	4.6 ± 1.6	0.8 ± 0.4	17.3 ± 1.4	0.32 ± 0.08	99.5 ± 0.2	2.3 ± 0.4	0.4 ± 0.2	26.8 ± 12.4	Nd	0.6 ± 0.1	4.4 ± 0.9	0.24 ± 0.05	3.3 ± 0.1
	10% glucose	84 ± 15	7.8 ± 2.0	6.4 ± 2.2	2.9 ± 1.5	17.4 ± 1.3	0.37 ± 0.09	98.3 ± 0.9	1.3 ± 0.4	0.3 ± 0.1	58.7 ± 12.3	Nd	0.7 ± 0.2	3.3 ± 0.7	0.18 ± 0.04	3.3 ± 0.1
	25% glucose	96 ± 18	8.5 ± 1.0	6.8 ± 2.4	3.9 ± 1.9	17.0 ± 1.2	0.41 ± 0.04	97.7 ± 1.1	1.7 ± 0.5	0.5 ± 0.2	47.0 ± 15.7	Nd	0.8 ± 0.3	3.8 ± 0.8	0.18 ± 0.04	3.2 ± 0.1
	10% sucrose	84 ± 15	7.8 ± 2.0	6.2 ± 2.2	3.1 ± 1.5	17.4 ± 1.3	0.37 ± 0.09	98.2 ± 0.9	1.5 ± 0.5	0.6 ± 0.3	52.0 ± 15.6	Nd	0.9 ± 0.3	3.0 ± 0.6	0.18 ± 0.04	3.3 ± 0.1
	25% sucrose	96 ± 16	9.3 ± 1.2	7.6 ± 2.7	8.1 ± 4.0	18.5 ± 0.8	0.44 ± 0.03	95.3 ± 2.4	1.9 ± 0.6	0.7 ± 0.3	44.0 ± 14.3	Nd	1.0 ± 0.3	3.5 ± 0.7	0.18 ± 0.04	3.3 ± 0.1
	10% lactose	120 ± 22	6.0 ± 1.5	4.9 ± 1.7	3.6 ± 1.8	9.4 ± 0.7	0.28 ± 0.07	97.9 ± 1.1	2.6 ± 0.3	0.4 ± 0.2	18.8 ± 9.2	Nd	0.7 ± 0.1	3.5 ± 0.7	0.39 ± 0.08	3.7 ± 0.1
	25% lactose	75 ± 14	4.9 ± 1.2	3.2 ± 1.1	2.9 ± 1.4	12.2 ± 0.9	0.23 ± 0.06	98.3 ± 0.8	2.9 ± 0.1	0.4 ± 0.2	9.4 ± 3.8	Nd	0.6 ± 0.1	2.9 ± 0.6	0.21 ± 0.04	3.3 ± 0.1
	10% trehalose	96 ± 17	4.1 ± 1.1	2.5 ± 0.9	1.3 ± 0.6	8.1 ± 0.6	0.19 ± 0.05	99.2 ± 0.4	1.5 ± 0.5	0.3 ± 0.1	53.6 ± 15.1	Nd	0.5 ± 0.2	2.6 ± 0.5	0.24 ± 0.05	3.3 ± 0.1
	25% trehalose	96 ± 18	7.3 ± 1.9	6.0 ± 2.1	2.8 ± 1.4	14.4 ± 1.0	0.35 ± 0.09	98.3 ± 0.8	2.7 ± 0.2	0.6 ± 0.3	14.9 ± 6.0	Nd	1.0 ± 0.1	3.9 ± 0.8	0.21 ± 0.04	3.2 ± 0.1
	10% glycerol	98 ± 19	4.5 ± 1.2	15.0 ± 5.3	1.0 ± 0.5	8.7 ± 0.6	0.21 ± 0.05	99.4 ± 0.3	1.3 ± 0.4	0.2 ± 0.1	60.9 ± 12.7	Nd	0.3 ± 0.1	2.7 ± 0.6	0.27 ± 0.06	3.6 ± 0.1
	25% glycerol	120 ± 23	5.8 ± 1.5	36.1 ± 12.8	2.1 ± 1.0	9.0 ± 0.6	0.27 ± 0.07	98.8 ± 0.6	1.9 ± 0.6	0.3 ± 0.1	41.0 ± 19.2	Nd	0.2 ± 0.1	3.2 ± 0.7	0.27 ± 0.06	3.6 ± 0.1
	50% glycerol	144 ± 27	5.4 ± 1.4	47.4 ± 16.8	1.3 ± 0.6	7.1 ± 0.5	0.25 ± 0.06	99.2 ± 0.4	1.8 ± 0.6	0.4 ± 0.2	43.1 ± 18.5	Nd	0.4 ± 0.1	4.2 ± 0.9	0.42 ± 0.09	3.7 ± 0.1
	Grape Must	75 ± 15	5.9 ± 1.5	3.7 ± 1.3	0.6 ± 0.3	14.8 ± 0.9	0.27 ± 0.07	99.7 ± 0.2	2.1 ± 0.5	0.3 ± 0.1	33.0 ± 14.2	Nd	0.8 ± 0.2	3.0 ± 0.6	0.33 ± 0.07	3.3 ± 0.1
	No Cryoprotectant	120 ± 22	5.9 ± 1.5	3.0 ± 1.0	2.0 ± 1.0	9.3 ± 0.7	0.28 ± 0.07	98.8 ± 0.6	1.4 ± 0.5	0.6 ± 0.3	55.1 ± 14.6	0.8 ± 0.4	0.4 ± 0.1	3.8 ± 0.8	0.63 ± 0.13	3.8 ± 0.1
Immob. cells on DCM	10% fructose	93 ± 7	9.0 ± 1.4	6.5 ± 1.3	5.3 ± 1.4	18.2 ± 1.6	0.42 ± 0.05	96.9 ± 0.9	2.3 ± 0.6	0.7 ± 0.2	30.1 ± 13.9	Nd	1.4 ± 0.6	3.5 ± 0.3	0.24 ± 0.05	3.5 ± 0.1
	25% fructose	75 ± 5	6.9 ± 1.1	4.8 ± 0.9	9.9 ± 2.5	17.5 ± 1.5	0.34 ± 0.05	94.2 ± 1.5	1.7 ± 0.4	0.4 ± 0.1	48.0 ± 14.0	Nd	0.9 ± 0.4	3.2 ± 0.3	0.21 ± 0.04	3.5 ± 0.1
	10% glucose	96 ± 7	9.7 ± 1.0	5.9 ± 1.2	1.3 ± 0.3	19.2 ± 0.5	0.44 ± 0.03	99.3 ± 0.2	3.0 ± 0.3	0.3 ± 0.1	9.7 ± 3.8	Nd	1.1 ± 0.5	3.9 ± 0.4	0.27 ± 0.05	3.5 ± 0.1
	25% glucose	93 ± 7	10.0 ± 0.4	7.8 ± 1.5	5.0 ± 1.3	20.4 ± 0.6	0.47 ± 0.01	97.1 ± 0.8	2.8 ± 0.4	0.8 ± 0.3	16.7 ± 8.2	Nd	1.8 ± 0.9	3.9 ± 0.4	0.24 ± 0.04	3.5 ± 0.1
	10% sucrose	96 ± 6	10.4 ± 0.1	8.1 ± 1.6	1.6 ± 0.4	21.2 ± 0.9	0.47 ± 0.01	99.1 ± 0.2	2.7 ± 0.2	0.8 ± 0.3	20.5 ± 6.7	Nd	1.5 ± 0.6	4.1 ± 0.4	0.21 ± 0.04	3.5 ± 0.1
	25% sucrose	93 ± 5	9.7 ± 0.9	7.0 ± 1.4	4.0 ± 1.0	21.3 ± 0.4	0.45 ± 0.02	97.7 ± 0.7	2.1 ± 0.5	0.4 ± 0.1	37.9 ± 12.3	Nd	1.4 ± 0.7	3.6 ± 0.4	0.27 ± 0.05	3.5 ± 0.1
	10% lactose	96 ± 7	8.1 ± 1.3	4.5 ± 0.9	2.3 ± 0.6	16.0 ± 1.4	0.38 ± 0.06	98.7 ± 0.3	2.5 ± 0.3	0.4 ± 0.1	21.9 ± 9.9	Nd	1.2 ± 0.6	3.5 ± 0.3	0.18 ± 0.04	3.4 ± 0.1
	25% lactose	96 ± 7	6.9 ± 1.1	3.9 ± 0.8	2.7 ± 0.7	13.5 ± 1.2	0.32 ± 0.05	98.4 ± 0.4	2.8 ± 0.2	0.3 ± 0.1	12.5 ± 6.2	Nd	0.9 ± 0.4	3.2 ± 0.3	0.21 ± 0.04	3.4 ± 0.1
	10% trehalose	82 ± 6	7.6 ± 1.2	4.3 ± 0.8	1.7 ± 0.4	17.4 ± 1.5	0.35 ± 0.05	99.0 ± 0.3	2.9 ± 0.2	0.3 ± 0.1	10.7 ± 5.1	Nd	1.0 ± 0.5	3.5 ± 0.3	0.18 ± 0.04	3.4 ± 0.1
	25% trehalose	82 ± 6	8.4 ± 1.3	4.8 ± 1.0	1.7 ± 0.4	19.3 ± 1.6	0.39 ± 0.06	99.0 ± 0.3	2.8 ± 0.2	0.3 ± 0.1	13.7 ± 6.1	Nd	0.9 ± 0.4	3.6 ± 0.4	0.15 ± 0.03	3.4 ± 0.1
	10% glycerol	96 ± 7	8.7 ± 1.4	11.5 ± 2.3	1.5 ± 0.4	17.2 ± 1.5	0.41 ± 0.06	99.1 ± 0.2	3.1 ± 0.1	0.4 ± 0.1	2.9 ± 1.4	Nd	1.1 ± 0.5	3.6 ± 0.4	0.18 ± 0.04	3.4 ± 0.1
	25% glycerol	93 ± 7	7.5 ± 1.2	28.3 ± 5.6	1.6 ± 0.4	15.2 ± 1.3	0.35 ± 0.05	99.0 ± 0.2	2.3 ± 0.4	0.2 ± 0.1	29.3 ± 12.0	Nd	0.8 ± 0.4	2.9 ± 0.3	0.24 ± 0.05	3.5 ± 0.1
	50% glycerol	93 ± 8	7.3 ± 1.1	16.1 ± 3.0	1.6 ± 0.4	14.9 ± 1.1	0.34 ± 0.05	99.1 ± 0.2	2.5 ± 0.3	0.2 ± 0.1	22.7 ± 9.8	Nd	0.9 ± 0.4	3.2 ± 0.3	0.18 ± 0.04	3.5 ± 0.1
	Grape Must	93 ± 8	8.9 ± 1.4	7.7 ± 1.5	6.5 ± 1.7	18.1 ± 1.3	0.42 ± 0.05	96.2 ± 1.1	2.3 ± 0.5	0.5 ± 0.2	30.9 ± 11.7	Nd	2.1 ± 0.9	3.3 ± 0.3	0.27 ± 0.05	3.4 ± 0.1
	No Cryoprotectant	90 ± 6	8.0 ± 1.2	5.8 ± 1.2	3.3 ± 0.8	16.8 ± 1.4	0.38 ± 0.06	98.1 ± 0.5	1.8 ± 0.5	1.6 ± 0.5	43.2 ± 14.5	1.4 ± 0.5	0.6 ± 0.3	4.7 ± 0.5	0.66 ± 0.13	3.7 ± 0.1
Immob. cells on grape skins	10% fructose	160 ± 11	9.5 ± 0.9	5.6 ± 1.1	5.2 ± 1.3	11.3 ± 0.3	0.45 ± 0.03	97.0 ± 0.9	2.4 ± 0.5	0.4 ± 0.1	27.0 ± 13.4	Nd	0.7 ± 0.3	4.4 ± 0.4	0.36 ± 0.07	3.7 ± 0.1
	25% fructose	146 ± 10	7.7 ± 1.2	4.7 ± 0.9	8.1 ± 2.0	9.9 ± 0.8	0.37 ± 0.05	95.3 ± 1.2	2.0 ± 0.5	0.3 ± 0.1	37.3 ± 14.2	Nd	0.6 ± 0.3	4.4 ± 0.4	0.36 ± 0.07	3.7 ± 0.1
	10% glucose	160 ± 11	8.4 ± 1.3	5.3 ± 1.1	3.7 ± 0.9	10.0 ± 0.9	0.40 ± 0.06	97.8 ± 0.6	2.3 ± 0.4	0.4 ± 0.1	26.9 ± 12.4	Nd	0.6 ± 0.3	4.1 ± 0.4	0.33 ± 0.07	3.7 ± 0.1
	25% glucose	158 ± 11	9.6 ± 1.0	6.7 ± 1.3	5.4 ± 1.4	11.5 ± 0.3	0.45 ± 0.03	96.9 ± 0.9	2.8 ± 0.3	0.8 ± 0.3	14.5 ± 6.0	Nd	0.6 ± 0.3	4.1 ± 0.4	0.36 ± 0.07	3.7 ± 0.1
	10% sucrose	142 ± 10	4.8 ± 0.8	3.0 ± 0.6	3.8 ± 1.0	6.5 ± 0.6	0.23 ± 0.03	97.8 ± 0.6	1.3 ± 0.3	0.2 ± 0.1	60.4 ± 10.6	Nd	0.6 ± 0.3	3.0 ± 0.3	0.30 ± 0.06	3.7 ± 0.1
	25% sucrose	146 ± 8	7.9 ± 1.2	5.1 ± 1.0	6.3 ± 1.6	10.2 ± 1.0	0.38 ± 0.06	96.3 ± 0.9	1.7 ± 0.4	0.2 ± 0.1	48.3 ± 11.7	Nd	0.2 ± 0.1	3.8 ± 0.4	0.33 ± 0.07	3.7 ± 0.1
	10% lactose	193 ± 14	7.1 ± 1.1	5.2 ± 1.0	3.4 ± 0.9	6.9 ± 0.6	0.33 ± 0.05	98.0 ± 0.5	2.6 ± 0.3	0.6 ± 0.2	18.8 ± 9.2	Nd	0.5 ± 0.2	3.9 ± 0.4	0.36 ± 0.07	3.7 ± 0.1
	25% lactose	196 ± 14	8.5 ± 1.3	5.4 ± 1.1	3.6 ± 0.9	8.2 ± 0.7	0.40 ± 0.06	97.9 ± 0.5	2.7 ± 0.2	0.3 ± 0.1	15.6 ± 7.2	Nd	0.6 ± 0.3	4.1 ± 0.4	0.36 ± 0.07	3.7 ± 0.1
	10% trehalose	146 ± 10	9.8 ± 0.4	6.6 ± 1.3	8.9 ± 2.3	12.8 ± 0.4	0.46 ± 0.01	95.0 ± 1.3	2.9 ± 0.2	0.8 ± 0.3	14.5 ± 6.0	Nd	0.8 ± 0.3	4.7 ± 0.5	0.33 ± 0.06	3.8 ± 0.1
	25% trehalose	158 ± 11	8.5 ± 1.3	5.7 ± 1.1	4.5 ± 1.2	10.1 ± 0.9	0.40 ± 0.06	97.3 ± 0.7	2.6 ± 0.3	0.5 ± 0.2	18.9 ± 8.0	Nd	0.6 ± 0.2	4.1 ± 0.4	0.33 ± 0.07	3.8 ± 0.1
	10% glycerol	166 ± 12	7.2 ± 1.1	10.2 ± 2.0	3.7 ± 0.9	8.2 ± 0.7	0.34 ± 0.05	97.8 ± 0.6	2.6 ± 0.3	0.7 ± 0.2	19.8 ± 7.9	Nd	0.6 ± 0.3	4.7 ± 0.5	0.39 ± 0.08	3.6 ± 0.1
	25% glycerol	170 ± 12	7.2 ± 1.1	14.1 ± 2.8	3.3 ± 0.8	8.1 ± 0.7	0.34 ± 0.05	98.0 ± 0.5	2.3 ± 0.3	0.6 ± 0.2	27.2 ± 10.3	Nd	0.6 ± 0.3	4.1 ± 0.4	0.36 ± 0.07	3.6 ± 0.1
	50% glycerol	160 ± 14	7.2 ± 1.1	23.8 ± 4.4	2.4 ± 0.6	8.6 ± 0.6	0.34 ± 0.05	98.6 ± 0.4	2.2 ± 0.5	0.4 ± 0.1	30.5 ± 14.7	Nd	0.6 ± 0.3	4.2 ± 0.4	0.42 ± 0.08	3.7 ± 0.1
	Grape Must	158 ± 13	8.6 ± 1.3	5.6 ± 1.1	3.9 ± 1.0	10.3 ± 0.7	0.41 ± 0.06	97.7 ± 0.6	2.4 ± 0.3	0.3 ± 0.1	25.7 ± 9.5	Nd	0.6 ± 0.3	4.4 ± 0.4	0.36 ± 0.07	3.8 ± 0.1
	No Cryoprotectant	156 ± 11	9.2 ± 1.4	6.7 ± 1.3	5.7 ± 1.5	11.1 ± 1.0	0.43 ± 0.05	96.7 ± 0.9	2.8 ± 0.3	0.8 ± 0.3	14.1 ± 6.1	0.1 ± 0.1	0.6 ± 0.3	5.0 ± 0.5	0.51 ± 0.10	3.7 ± 0.1
*F*-values																
Nature of kefir culture		111.86 **	26.59 **	13.95 **	18.06 **	280.23 **	30.79 **	16 **	9.35 **	1.20	9.98 **	5.62 **	18.08 **	15.11 **	61.03 **	166.2 **
Cryoprotectant used		4.05 **	2.26 *	25.06 **	16.04 **	22.36 **	2.89 **	14 **	2.95 **	7.82 **	3.88 **	54.90 **	1.18	1.27	19.86 **	11.2 **
Interaction		3.08 **	1.64 *	6.57 **	6.87 **	13.09 **	1.81 *	6 **	2.38 **	2.53 **	2.76 **	5.62 **	0.68	1.41	2.73 **	9.0 **

Nd: Not detected; * *p* < 0.05, ** *p* < 0.01; † Initial grape must malic acid content: 3.2 ± 0.2 g/L.

**Table 2:** Fermentation parameters of low alcohol wines produced by repeated batch fermentations at 5–30 °C using wet or freeze-dried kefir culture.

Nature of Kefir Culture	°C	N°	Fermentation Time (h)		Ethanol Concentration (% vol)		Glycerol (g/L)		Residual Sugars (g/L)		Ethanol Productivity [g/(Ld)]		Ethanol Production Yield		Conversion (%)	
State of the Cells:			W	Fd	W	Fd	W	Fd	W	Fd	W	Fd	W	Fd	W	Fd
Free cells	30	1–3	154–165	110–240	9.6–10.5	6.3–9.0	5.7–6.4	5.0–7.1	2.0–10.5	3.7–5.5	11.0–12.8	7.0–15.5	0.45–0.47	0.30–0.43	94.1–98.9	96.8–97.8
	20	4–8	155–250	90–135	10.2–10.5	7.4–10.1	6.4–7.4	4.4–9.6	1.7–6.6	2.5–7.7	8.0–12.9	10.8–16.4	0.47–0.48	0.35–0.46	96.3–99.0	95.5–98.5
	5	9–11	950–1078	1500–4000	7.3–10.2	7.8–9.0	5.1–6.2	5.7–7.6	7.7–15.5	1.9–5.0	1.3–1.9	0.4–1.0	0.36–0.47	0.37–0.43	91.3–95.7	97.1–98.9
Immob. cells on apple pieces	30	1–3	50–70	72–120	7.1–9.5	5.9–8.2	4.7–6.8	3.0–5.9	2.8–5.2	2.0–4.4	19.4–30.0	9.3–20.0	0.33–0.45	0.28–0.39	96.9–98.3	97.4–98.8
	20	4–8	94–115	72–100	7.3–10.5	7.6–10.3	5.8–7.6	6.7–9.9	3.4–9.1	1.8–5.6	14.7–21.4	16.0–23.2	0.36–0.49	0.36–0.47	94.6–98.0	96.8–98.9
	5	9–11	940–1220	816–1300	6.6–8.3	7.7–10.5	4.3–6.0	6.5–8.4	8.1–10.3	1.9–8.3	1.0–1.4	1.4–2.1	0.33–0.40	0.36–0.47	94.0–95.2	95.1–98.9
Immob. cells on DCM	30	1–3	48–140	70–90	7.0–10.0	7.2–9.0	5.7–7.3	5.8–7.8	2.2–7.4	3.3–4.8	9.5–39.5	16.9–24.4	0.33–0.46	0.34–0.43	95.6–98.7	97.2–98.1
	20	4–8	53–105	72–100	8.2–10.3	8.6–10.0	7.1–8.6	6.6–8.8	3.2–9.7	2.0–9.7	18.7–31.6	16.5–22.6	0.40–0.47	0.42–0.47	94.3–98.2	94.3–98.8
	5	9–11	590–740	480–650	7.1–9.6	7.0–9.1	6.2–8.4	5.6–8.3	6.3–10.5	1.6–2.2	1.9–2.5	2.2–2.8	0.35–0.46	0.33–0.43	93.8–96.3	98.7–99.1
Immob. cells on grape skins	30	1–3	72–94	120–156	5.9–8.8	9.2–9.9	5.5–7.9	6.7–7.6	5.3–7.2	5.1–6.4	15.6–20.8	11.2–14.7	0.29–0.42	0.44–0.47	95.8–96.9	96.2–97.0
	20	4–8	75–120	90–175	7.1–8.7	9.7–10.5	6.2–9.1	7.7–10.8	4.1–7.6	1.2–8.1	13.7–19.1	10.8–21.1	0.35–0.41	0.46–0.47	95.5–97.6	95.4–99.3
	5	9–11	670–780	740–910	4.5–7.3	10.0–10.5	4.2–5.6	9.1–10.2	8.3–11.8	1.4–5.0	1.3–1.9	2.2–2.6	0.22–0.36	0.46–0.47	93.0–95.1	97.2–99.2
*F*-values																
State of the cells			7.44 **		2.59		14.71 **		33.51 **		4.61 *		1.73		33.4 **	
Nature of kefir culture			15.77 **		2.76 *		6.50 **		0.46		13.60 **		2.17		0.4	
Fermentation temperature			154.32 **		7.96 **		11.38 **		3.85 *		130.34 **		7.77 **		3.9 *	
All interactions			7.49 **		0.55		2.02		0.81		1.46		0.68		0.8	

N°: Number of repeated fermentation batches; W: Wet cells, Fd: Freeze-dried cells; * *p* < 0.05, ** *p* < 0.01.

**Table 3:** Organic acids profile and enological parameters of low alcohol wines produced by repeated batch fermentations at 5–30 °C using wet or freeze-dried kefir culture.

Nature of Kefir Culture	°C	N°	Malic Acid † (g/L)		Lactic Acid (g/L)		Malic Acid Conversion (%)		Acetic Acid (g/L)		Citric Acid (g/L)		Propionic Acid (g/L)		Total Acidity (g Tartaric/L)		Volatile Acidity (g Acetic/L)		pH	
State of the Cells:			W	Fd	W	Fd	W	Fd	W	Fd	W	Fd	W	Fd	W	Fd	W	Fd	W	Fd
Free cells	30	1–3	2.1–2.5	1.7–2.5	0.4–0.5	0.3–1.0	25.1–36.9	23.1–45.6	0.6–0.8	0.5–1.0	0.3–0.4	0.5–0.8	<0.1–0.1	Nd	3.6–3.8	3.2–4.4	0.39–0.45	0.66–0.90	4.0–4.1	3.7–3.8
	20	4–8	2.4–2.5	2.1–2.8	0.4–0.7	0.2–0.5	25.0–29.5	17.0–34.2	0.6–1.1	0.1–1.1	0.2–0.3	0.6–0.8	<0.1	0.0–0.1	3.8–4.2	3.9–4.7	0.36–0.87	0.69–0.93	4.0	3.7–3.8
	5	9–11	1.7–2.3	1.1–2.3	0.3–0.4	0.4–0.5	33.2–47.4	27.8–64.3	1.7–1.9	1.4–2.2	<0.1–0.2	0.5–0.7	<0.1–0.1	0.0–0.1	3.9–4.1	3.2–4.2	0.96–1.05	1.38–1.68	4.2	3.6–4.1
Immob. cells on apple pieces	30	1–3	1.7–2.6	1.4–2.1	1.2–1.8	0.6–1.7	18.4–47.0	34.4–55.1	<0.1–0.9	0.8–1.4	0.7–0.8	0.4–0.9	Nd	0.0–0.1	3.6–4.2	3.8–5.1	0.45–0.78	0.63–1.29	3.7–3.8	3.6–3.9
	20	4–8	2.2–2.9	2.3–2.8	0.7–0.9	0.2–0.9	14.8–30.6	11.0–28.3	0.3–0.5	0.1–0.2	0.2–0.3	0.6–1.3	Nd	Nd	2.6–4.8	4.4–5.4	0.36–0.45	0.57–0.72	3.8–4.0	3.6–3.7
	5	9–11	1.5–2.1	2.1–2.5	0.2–0.6	0.2–0.5	34.8–52.0	21.4–35.0	0.2–1.0	1.4–2.2	0.2–0.7	0.6	Nd	0.0–0.1	3.5–3.9	3.9–4.7	0.75–0.87	1.17–1.62	4.0–4.1	3.9–4.1
Immob. cells on DCM	30	1–3	1.3–2.6	1.8–2.8	1.4–4.1	0.7–1.6	22.5–59.3	13.6–43.2	0.1–2.0	0.1–1.4	0.4–0.5	0.5–0.7	0.0–<0.1	0.0–0.1	3.9–5.7	4.2–4.7	0.42–1.89	0.57–0.66	3.6–3.8	3.6–3.7
	20	4–8	2.2–2.6	2.5–2.8	0.6–0.8	0.3–0.7	20.2–30.3	14.0–23.0	<0.1–0.4	0.0–0.1	0.2–0.8	0.4–0.5	Nd	0.0–0.1	3.5–3.8	3.6–4.4	0.39–0.63	0.48–0.72	3.8–3.9	3.7–3.9
	5	9–11	1.8–2.4	1.9–2.5	0.5–0.6	0.2–0.5	26.3–43.5	20.4–39.8	0.1–0.2	0.1–1.0	0.2	0.5–0.6	Nd	Nd	3.2–3.5	3.3–4.1	0.72–0.87	0.63–1.14	4.1	4.0
Immob. cells on grape skins	30	1–3	1.9–2.8	2.8–2.9	0.7–1.1	0.8–0.9	12.4–39.9	9.0–11.3	0.3–0.5	0.1	0.3–0.7	0.6–0.8	<0.1–0.1	Nd	4.2–4.4	5.0–5.1	0.42–0.45	0.51–0.63	3.7	3.7–3.8
	20	4–8	1.8–2.8	1.6–2.9	0.5–0.9	0.6–0.8	12.3–44.5	10.6–54.0	0.3–0.5	0.0–0.2	0.2–0.3	0.2–0.9	<0.1–0.1	0.0–0.1	3.8–4.5	4.4–5.9	0.45–0.51	0.45–0.72	3.7–3.8	3.4–3.8
	5	9–11	1.4–1.8	1.1–1.2	0.3–0.4	0.5–0.7	42.7–56.3	63.4–67.3	1.0–1.2	0.2–1.7	0.1–0.2	0.6–1.6	<0.1	0.0–0.1	3.0–3.8	4.4–5.3	0.96–1.14	0.87–1.20	4.1	3.5–3.7
*F*-values																				
State of the cells			0.07		7.41 **		0.34		0.11		71.06 **		6.18 *		28.29 **		19.76 **		88.8 **	
Nature of kefir culture			0.56		6.87 **		0.60		15.12 **		3.20 *		6.11 **		5.08 **		2.88 *		17.6 **	
Fermentation temperature			20.96 **		28.92 **		20.47 **		33.44 **		2.80		2.71		5.90 **		51.29 **		59.4 **	
All interactions			1.34		1.57		1.46		1.47		3.88 **		3.70 **		0.72		1.52		4.4 **	

N°: Number of repeated fermentation batches; W: Wet cells, Fd: Freeze-dried cells; Nd: Not detected; * *p* < 0.05, ** *p* < 0.01; † Initial grape must malic acid content: 3.2 ± 0.2 g/L.

**Table 4:** Μajor volatiles of low alcohol wines produced by repeated batch fermentations at 5–30 °C using wet or freeze-dried kefir culture.

Nature of Kefir Culture	°C	N°	Acetaldehyde (mg/L)		Ethyl Acetate (mg/L)		1-Propanol (mg/L)		Isobutanol (mg/L)		1-Hexanol (mg/L)		Amyl Alcohol (mg/L)		Isoamyl Alcohol (mg/L)		Methanol (mg/L)	
State of the Cells:			W	Fd	W	Fd	W	Fd	W	Fd	W	Fd	W	Fd	W	Fd	W	Fd
Free cells	30	1–3	40–54	8–13	2–9	0–3	28–39	10–18	36–49	17–34	2–11	0–2	15–21	6–12	53–73	21–44	10–15	0–4
	20	4–8	60–88	10–17	3–10	0–3	34–49	12–23	44–64	18–39	0–3	1–3	19–29	8–15	71–104	36–63	5–21	3–5
	5	9–11	32–65	10–13	2–4	3–4	9–31	5–6	10–50	7–8	0–3	Nd	6–24	2–4	19–83	8–14	4–9	4–9
Immob. cells on apple pieces	30	1–3	7–13	5–41	4–5	0–30	24–37	9–12	44–58	21–32	5–11	1–5	16–21	6–8	60–79	23–33	5–16	5–10
	20	4–8	14–21	8–14	3–7	0–6	42–77	16–33	55–107	37–84	3–6	1–3	20–39	7–16	84–159	38–82	8–19	0–17
	5	9–11	14–35	12–39	2–9	4–5	37–67	11–16	24–50	9–20	1–3	0–1	10–23	5–6	43–88	19–32	6–17	0–8
Immob. cells on DCM	30	1–3	17–29	6–9	4–7	11–36	19–58	11–15	46–90	24–29	8–36	1–3	17–45	7–8	64–171	25–33	5–12	Nd
	20	4–8	17–37	2–30	3–8	3–14	31–51	4–14	41–62	6–26	2–4	0–1	17–29	2–8	67–110	10–37	6–17	Nd
	5	9–11	20–25	3–14	0–9	4–27	30–49	4–29	34–46	4–14	2	Nd	17–23	1–9	64–86	6–36	6–16	Nd
Immob. cells on grape skins	30	1–3	11–20	22–32	4–8	3–9	27–63	43–82	48–134	70–132	3–6	2–4	12–34	19–37	48–131	88–164	4–7	5–15
	20	4–8	15–29	14–27	2–10	5–13	33–69	45–74	49–102	41–75	2–5	2–3	16–34	21–34	67–143	97–159	5–28	7–24
	5	9–11	19–36	25–41	3	5–8	25–49	54–90	17–34	29–46	0–1	Nd	9–16	14–19	31–61	59–86	3–6	17–32
*F*-values																		
State of the cells	-	-	58.92 **		4.92 *		37.28 **		37.58 **		22.10 **		71.49 **		48.35 **		10.99 **	
Nature of kefir culture	-	-	22.05 **		5.14 **		36.09 **		19.82 **		1.38		7.70 **		13.76 **		7.65 **	
Fermentation temperature	-	-	2.23		1.54		3.54 *		27.08 **		15.09 **		13.01 **		17.65 **		3.31 *	
All interactions	-	-	0.99		0.81		1.24		0.33		1.99		0.37		0.37		2.16	

N°: Number of repeated fermentation batches; W: Wet cells, Fd: Freeze-dried cells; Nd: Not detected; * *p* < 0.05, ** *p* < 0.01.

**Table 5:** Minor volatile compounds (mg/L) identified in low alcohol wines produced by wet or freeze-dried kefir culture at 5–30 °C using the headspace solid-phase microextraction gas chromatography–mass spectrometry (HS-SPME/GC-MS) analysis. Volatiles were semi-quantified using 4-methyl-2-pentanol as the internal standard.

°C	Nature of Kefir Culture	State of the Cells	Esters	Organic Acids	Alcohols	Carbonyl Compounds	Miscellaneous Compounds	Total Volatiles
30	Free cells	W	54.0–104.6	0.4–4.4	16.2–39.4	0.0–0.4	4.3–7.2	75.0–156.0
		Fd	28.8–33.3	0.0–1.6	41.6–45.2	0.0–0.7	3.4–4.0	77.5–80.6
	Immob. cells on apple pieces	W	60.6–96.7	3.2–3.8	40.5–60.6	0.3–0.7	2.4–5.5	114.0–147.1
		Fd	19.9–28.2	Nd	18.9–102.6	Nd	1.3–4.7	40.1–132.0
	Immob. cells on DCM	W	48.8–81.8	0.3–2.2	18.7–54.3	0.0–0.3	3.0–3.9	71.1–132.9
		Fd	38.1–70.8	0.0–0.5	30.4–42.7	0.1–0.2	1.9–3.0	70.7–117.1
	Immob. cells on grape skins	W	42.9–165.3	0.0–1.4	27.1–52.0	0.0–0.1	0.6–2.1	88.1–220.7
		Fd	74.6–129.2	1.0–8.8	51.6–76.9	0.5–1.1	3.2–8.5	137.9–224.4
20	Free cells	W	84.0–181.9	0.0–3.3	28.8–52.4	0.0–0.2	3.8–8.4	130.2–221.6
		Fd	28.5–77.8	Nd	25.9–43.6	Nd	3.3–8.1	60.8–129.6
	Immob. cells on apple pieces	W	60.6–205.1	1.2–3.4	24.4–51.1	0.0–0.3	3.2–4.6	116.3–254.4
		Fd	57.0–244.4	0.0–5.6	32.2–83.4	0.0–0.2	4.7–10.1	105.2–337.8
	Immob. cells on DCM	W	36.3–143.3	0.0–3.1	30.3–53.9	Nd	2.1–3.6	91.5–188.3
		Fd	90.7–200.7	0.0–0.9	17.6–37.3	Nd	1.8–6.2	122.7–242.3
	Immob. cells on grape skins	W	38.6–314.5	0.1–4.9	34.2–55.9	0.0–0.5	2.3–4.3	97.0–368.1
		Fd	67.3–173.7	1.2–3.5	40.5–68.0	0.0–0.5	3.3–5.7	127.4–239.2
5	Free cells	W	60.3–75.0	1.1–1.6	9.7–13.2	0.1–0.2	3.8–7.7	82.6–90.1
		Fd	16.3–78.8	0.0–0.3	8.9–33.6	0.0–0.1	2.0–12.3	27.8–124.7
	Immob. cells on apple pieces	W	61.1–73.4	1.3–2.5	10.7–28.7	<0.1–0.1	3.6–3.8	90.3–101.9
		Fd	91.5–215.2	0.0–2.7	25.4–47.1	Nd	3.6–12.6	120.6–274.9
	Immob. cells on DCM	W	93.9–127.3	4.8–10.6	18.6–27.8	<0.1–0.2	3.3–4.1	126.7–164.3
		Fd	53.4–283.4	Nd	23.8–73.9	Nd	2.6–8.0	96.1–365.3
	Immob. cells on grape skins	W	58.9–141.5	0.7–5.1	10.0–26.0	<0.1–0.2	2.2–4.1	71.9–171.1
		Fd	31.8–86.8	0.0–1.9	25.9–35.3	0.0–0.7	3.7–4.1	64.1–126.8
*F*-values								
State of the cells			2.69	18.70 **	9.91 **	1.03	4.19 *	0.55
Nature of kefir culture			3.54 *	2.93 *	4.91 **	8.42 **	2.82 *	4.47 **
Fermentation temperature			10.20 **	0.14	14.54 **	15.69 **	2.60	8.72 **
All interactions			1.40	4.00 **	0.84	6.50 **	1.34	1.19

W: Wet cells, Fd: Freeze-dried cells; Nd: Not detected; * *p* < 0.05, ** *p* < 0.01.

**Table 6:** Sensory evaluation of low alcohol wines produced by wet and freeze-dried kefir culture at various temperatures (5–30 °C).

Fermentation Temperature (°C)	Low Alcohol Wine Sample	Quality Attribute					
		Aroma		Taste		Overall Quality	
State of the Cells:		W	Fd	W	Fd	W	Fd
30	Fr	3.4 ± 0.5	3.2 ± 0.8	3.2 ± 0.7	3.3 ± 0.9	3.0 ± 0.8	3.2 ± 0.7
	Ap	2.4 ± 0.5	2.8 ± 0.7	3.0 ± 0.7	3.3 ± 0.4	2.5 ± 0.8	2.7 ± 0.7
	DCM	3.3 ± 0.6	3.3 ± 0.6	4.0 ± 0.6	3.5 ± 0.5	3.9 ± 0.8	3.4 ± 0.7
	GS	2.2 ± 0.6	2.3 ± 0.6	3.3 ± 0.8	3.2 ± 0.6	3.0 ± 0.7	3.4 ± 0.9
20	Fr	3.4 ± 0.5	2.9 ± 0.7	3.5 ± 0.8	3.9 ± 0.5	2.5 ± 0.6	2.9 ± 0.9
	Ap	2.2 ± 0.6	2.0 ± 0.9	3.0 ± 0.7	2.5 ± 0.7	3.0 ± 0.7	2.0 ± 0.7
	DCM	2.3 ± 0.5	3.2 ± 0.7	3.5 ± 0.5	3.0 ± 0.9	2.2 ± 0.7	2.5 ± 0.9
	GS	2.3 ± 0.5	3.2 ± 0.7	3.2 ± 0.7	3.2 ± 0.9	2.8 ± 0.7	3.1 ± 0.8
5	Fr	3.0 ± 0.9	2.0 ± 0.8	3.5 ± 0.7	2.8 ± 0.7	2.9 ± 0.7	2.8 ± 0.7
	Ap	2.0 ± 0.5	2.5 ± 0.8	3.0 ± 0.7	3.3 ± 0.7	2.6 ± 0.5	3.2 ± 0.5
	DCM	2.0 ± 0.7	2.6 ± 0.7	2.3 ± 0.7	2.9 ± 0.8	2.9 ± 0.8	3.2 ± 0.7
	GS	2.2 ± 0.7	3.0 ± 0.7	3.0 ± 0.7	3.2 ± 0.8	3.2 ± 0.8	3.2 ± 0.8
Commercial wine		3.2 ± 0.8		2.7 ± 0.8		3.4 ± 0.9	
*F*-values							
State of the cells		6.25 *		0.21		0.70	
Nature of kefir culture		13.65 **		2.92 *		4.51 **	
Fermentation temperature		12.23 **		5.39 **		11.67 **	
All interactions		2.94 **		3.59 **		3.92 **	

Fr: low alcohol wine fermented by free kefir culture, Ap: low alcohol wine fermented by immobilized kefir culture on apple pieces, DCM: low alcohol wine fermented by immobilized kefir culture on DCM, GS: low alcohol wine fermented by immobilized kefir culture on grape skins; W: Wet cells, Fd: Freeze-dried cells; 0: unacceptable, 5: wonderful; * *p* < 0.05, ** *p* < 0.01.

## Supplementary Materials

The following are available online at https://www.mdpi.com/2304-8158/9/2/115/s1, Table S1: Minor volatile compounds (mg/L) identified in low alcohol wines produced by wet or freeze-dried kefir culture at various temperatures (5–30 °C) using the HS-SPME GC/MS analysis. Volatiles were semi-quantified using 4-methyl-2-pentanol as internal standard.
