# Supplementary material for: Freeze-Dried Immobilized Kefir Culture in Low Alcohol Winemaking
Source: Foods. 2020 Jan 21;9(2):115. doi: 10.3390/foods9020115 (PMC7073665; doi:10.3390/foods9020115)
Supplement: Supplementary file 1 [file foods-09-00115-s001.pdf]

Table S1. Minor volatile compounds (mg/L) identified in low alcohol wines produced by wet or freeze-dried kefir culture at various temperatures (5–30 °C) using the HS-SPME GC/MS analysis. Volatiles were semi–quantified using 4-methyl-2-pentanol as internal standard.

			30 °C								20 °C								5 °C							
**Compounds**	**KI**	***F* values**	**Free kefir culture**		**Kefir immob. on apple pieces**		**Kefir immob. on DCM**		**Kefir immob. on grape skins**		**Free kefir culture**		**Kefir immob. on apple pieces**		**Kefir immob. on DCM**		**Kefir immob. on grape skins**		**Free kefir culture**		**Kefir immob. on apple pieces**		**Kefir immob. on DCM**		**Kefir immob. on grape skins**	
**State of the cells:**			**W**	**Fd**	**W**	**Fd**	**W**	**Fd**	**W**	**Fd**	**W**	**Fd**	**W**	**Fd**	**W**	**Fd**	**W**	**Fd**	**W**	**Fd**	**W**	**Fd**	**W**	**Fd**	**W**	**Fd**
Esters																										
Ethyl acetate	<700		1.2–5.1	1.3–3.3	4.7–6.1	10.2–15.5	1.0–11.5	2.6–7.5	2.0–6.1	4.0–5.7	3.5–8.4	2.0–3.1	3.6–6.9	1.8–7.5	4.0–12.6	1.2–3.5	6.2–8.6	5.1–15.2	4.4–7.7	3.5–11.2	5.3–8.6	5.9–24.3	6.8–8.8	5.7–9.0	3.3–8.8	6.1–11.9
Ethyl propanoate	707		<0.1–0.1	0.0–0.2	<0.1–0.1	0.0–0.8	0.0–0.1	0.1	<0.1–0.3	0.2	0.2–0.3	0.0–0.1	0.0–0.3	0.0–1.6	0.0–0.4	0.0–0.1	0.2–0.4	0.1–0.8	0.2	0.0–0.1	0.1–0.8	0.2–0.6	0.4–0.5	0.0–0.2	0.5–1.3	0.3–0.7
Isobutyl acetate	745		0.0–<0.1	0.0–<0.1	0.0–0.1	0.0–0.2	<0.1–0.2	<0.1–0.1	0.1–0.3	0.2	0.0–0.1	Nd	0.1–0.3	0.0–0.1	0.0–0.2	0.0–0.1	0.2–0.3	0.0–0.3	0.0–<0.1	Nd	0.0–0.1	0.0–0.1	0.1	Nd	<0.1–0.1	0.1
Ethyl butyrate	803		0.3–0.4	0.0–0.2	0.3–0.4	0.0–0.2	0.1–0.6	<0.1–0.3	0.2–0.6	0.4–0.5	0.4–0.6	0.0–0.2	0.5–0.8	0.3–0.5	0.5–0.9	0.2–0.6	0.5–0.9	0.4–1.3	0.4–0.5	0.0–0.3	0.3–1.0	0.8–1.9	0.5–0.8	1.0–2.0	0.3–0.7	0.6–0.7
3-methylbutyl acetate (Isoamyl acetate)	867		0.9–1.3	0.0–1.1	3.6–4.8	0.6–1.4	1.5–4.6	0.5–1.0	2.6–8.6	6.1–8.3	3.7–5.8	0.4–1.1	7.0–12.5	1.0–2.0	4.1–10.3	0.7–1.7	5.9–14.0	5.5–20.6	1.2–1.6	0.0–0.7	3.6–5.8	0.5–1.3	4.9–6.8	0.4–2.3	2.3–5.7	4.1–8.7
2-methylbutyl acetate	869		0.0–0.1	0.0–0.1	0.3–0.4	0.0–0.3	0.1–0.3	0.0–0.1	0.2–0.6	0.4–0.5	0.0–0.3	Nd	0.5–0.8	0.0–0.2	0.0–0.6	0.0–0.2	0.3–0.7	0.3–1.0	0.1	0.0–0.1	0.0–0.3	0.0–0.1	0.3–0.4	Nd	0.2–0.3	0.2–0.4

Ethyl hexanoate	1002	3.7–4.8	0.8–3.6	4.6–6.1	0.3–1.7	2.7–5.3	1.5–7.7	2.9–15.6	7.7–12.1	8.0–14.2	2.4–8.1	9.2–21.2	8.0–14.4	4.0–18.4	10.8–22.3	4.2–32.7	5.4–15.7	5.5–11.7	0.0–10.5	3.9–7.0	6.6–11.8	8.4–18.0	3.0–33.1	8.1–18.3	3.2–8.9
Ethyl heptanoate	1126	Nd	Nd	Nd	Nd	Nd	Nd	Nd	Nd	0.0–0.2	Nd	0.0–0.3	Nd	Nd	Nd	0.0–0.3	Nd	Nd	Nd	0.1–0.2	Nd	Nd	Nd	0.1–0.2	Nd
Diethyl butanedioate	1191	0.1–0.8	0.0–0.5	0.5–0.8	0.0–0.1	0.4–1.4	0.3–0.7	0.2–0.3	0.6–1.7	0.0–0.6	0.0–0.4	0.5–0.8	0.0–1.0	0.0–0.5	0.0–0.6	0.5–1.5	0.0–0.6	0.7–0.8	0.0–0.4	0.4–0.8	0.0–0.6	0.4–0.8	0.0–0.6	0.5–1.3	0.0–0.5
Ethyl octanoate	1202	32.3–47.4	8.9–20.3	35.5–63.4	0.3–8.9	24.7–34.0	7.2–44.4	18.2–99.8	37.7–72.0	50.7–126.1	17.5–55.8	32.9–144.1	34.2–131.0	11.7–91.4	60.9–141.4	14.0–219.2	28.6–81.9	27.9–47.7	2.2–43.5	27.0–41.3	56.9–108.9	51.1–75.5	27.2–201.7	34.5–83.5	7.5–34.2
2-phenylethyl acetate	1263	0.6–3.8	0.4–7.1	2.0–2.9	0.3–1.8	11.4–24.4	7.5–18.9	1.2–4.2	1.7–3.1	1.2–1.8	0.2–0.5	1.1–3.1	0.4–0.9	1.5–2.9	0.4–2.0	0.8–3.7	2.1–10.2	1.1–1.7	0.0–1.8	0.6–0.8	0.0–0.3	2.0–2.6	0.2–1.9	0.7–2.0	0.8–2.1
Ethyl nonanoate	1307	0.0–0.1	Nd	0.1–0.2	Nd	0.0–0.2	Nd	0.0–<0.1	Nd	0.0–0.1	Nd	0.0–0.2	Nd	Nd	Nd	0.0–0.1	Nd	0.1	Nd	0.1	Nd	0.0–0.1	Nd	0.1–0.5	Nd
Citronellyl acetate	1360	Nd	Nd	Nd	Nd	Nd	Nd	0.0–<0.1	<0.1–0.1	Nd	Nd	0.0–0.1	Nd	Nd	Nd	0.0–0.1	Nd	0.0–<0.1	Nd	0.0–0.1	Nd	0.1	Nd	<0.1–0.1	0.0–0.1
Ethyl 9-decenoate	1390	2.5–4.8	1.4–3.2	1.0–1.7	Nd	1.3–2.8	0.3–1.2	0.7–3.0	2.3–4.6	3.6–9.7	1.4–2.8	0.7–7.9	0.7–11.6	0.6–5.3	1.9–5.7	1.8–18.6	3.0–7.9	5.4–11.2	0.9–19.5	4.0–10.4	11.8–43.8	7.6–11.7	0.0–19.2	5.6–19.6	2.6–10.9

Ethyl decanoate	1398		9.9–37.0	3.7–6.9	7.0–11.1	0.1–2.7	4.9–9.6	1.6–5.1	5.2–25.9	10.1–19.6	6.3–20.0	1.9–6.7	1.4–18.5	4.8–72.5	1.1–12.9	4.3–23.5	1.2–27.9	8.7–12.6	1.4–3.4	0.0–4.3	2.9–4.1	8.8–28.0	4.2–5.0	2.0–13.6	2.7–9.0	1.0–8.6
3-methylbutyl octanoate	1453		0.0–0.2	0.0–<0.1	0.1–0.2	Nd	0.1	Nd	0.0–0.2	0.1–0.4	0.0–0.2	Nd	0.0–0.6	0.0–0.9	0.0–0.1	0.0–0.3	0.0–0.3	Nd	Nd	Nd	0.0–0.1	0.0–0.2	0.0–0.3	Nd	<0.1–<0.1	0.0–<0.1
Ethyl dodecanoate	1595		<0.1–0.3	0.0–0.1	0.1–0.4	0.0–0.4	0.1–0.2	0.0–0.1	0.0–0.3	0.2–0.4	0.0–0.2	0.0–0.2	<0.1–0.9	0.0–1.5	Nd	0.1–1.2	0.2–0.5	0.2–3.4	0.0–<0.1	Nd	<0.1–0.1	0.0–1.1	<0.1	Nd	<0.1–<0.1	0.0–0.1
Ethyl-9-hexadecanoate	1975		Nd	Nd	Nd	Nd	Nd	Nd	Nd	Nd	Nd	Nd	Nd	0.0–4.3	Nd	0.0–<0.1	Nd	0.0–1.3	Nd	Nd	Nd	0.0–3.5	Nd	Nd	Nd	0.0–0.1
Ethyl hexadecanoate	1995		Nd	0–<0.1	Nd	<0.1–0.2	Nd	Nd	Nd	0.0–0.1	Nd	0.0–0.1	Nd	0.0–1.7	Nd	0.0–0.1	Nd	0.0–0.6	Nd	Nd	Nd	0.0–0.6	Nd	Nd	Nd	0.0–<0.1
Total esters			**54.0–104.6**	**28.8–33.3**	**60.6–96.7**	**19.9–28.2**	**48.8–81.8**	**38.1–70.8**	**42.9–165.3**	**74.6–129.2**	**84.0–181.9**	**28.5–77.8**	**60.6–205.1**	**57.0–244.4**	**36.3–143.3**	**90.7–200.7**	**38.6–314.5**	**67.3–173.7**	**60.3–75.0**	**16.3–78.8**	**61.1–73.4**	**91.5–215.2**	**93.9–127.3**	**53.4–283.4**	**58.9–141.5**	**31.8–86.8**
State of the cells		2.69																								
Nature of kefir culture		3.54*																								
Fermentation temperature		10.20**																								
All interactions		1.40																								
Organic acids																										
Hexanoic acid	1005		Nd	Nd	Nd	Nd	Nd	Nd	Nd	Nd	Nd	Nd	Nd	Nd	Nd	Nd	Nd	Nd	0.0–0.2	Nd	Nd	Nd	0.1–0.7	Nd	0.0–0.3	Nd

Octanoic acid	1198		0.4–2.1	0.0–1.2	2.8–3.2	Nd	0.3–1.9	0.0–0.5	0.0–1.3	1.0–6.1	0.0–2.8	Nd	1.1–3.1	0.0–3.8	0.0–3.1	0.0–0.7	0.1–4.4	1.2–2.9	0.9–1.5	0.0–0.3	1.1–2.2	0.0–2.3	4.1–8.9	Nd	0.4–3.0	0.0–1.6
Decanoic acid	1381		0.0–3.3	0.0–0.4	0.3–0.6	Nd	<0.1–0.3	Nd	0.0–0.9	<0.1–2.7	0.0–0.5	Nd	0.0–1.2	0.0–1.8	0.0–0.2	0.0–0.2	0.0–1.6	0.0–1.3	0.0–0.1	Nd	0.0–0.3	0.0–0.4	0.1–1.5	Nd	0.3–1.8	0.0–0.3
Total organic acids			**0.4–4.4**	**0.0–1.6**	**3.2–3.8**	**Nd**	**0.3–2.2**	**0.0–0.5**	**0.0–1.4**	**1.0–8.8**	**0.0–3.3**	**Nd**	**1.2–3.4**	**0.0–5.6**	**0.0–3.1**	**0.0–0.9**	**0.1–4.9**	**1.2–3.5**	**1.1–1.6**	**0.0–0.3**	**1.3–2.5**	**0.0–2.7**	**4.8–10.6**	**Nd**	**0.7–5.1**	**0.0–1.9**
State of the cells		18.70**																								
Nature of kefir culture		2.93*																								
Fermentation temperature		0.14																								
All interactions		4.00**																								
Alcohols																										
2-methyl-1-propanol (Isobutanol)	<700		0.3–0.5	1.0–3.0	0.8–1.6	0.6–7.4	0.3–1.3	1.4–1.8	1.3–1.9	1.6–1.8	0.4–1.5	0.8–1.6	0.4–1.6	1.0–4.8	0.6–2.3	0.3–1.4	1.0–2.7	1.3–2.4	0.1–0.2	0.0–0.3	0.0–0.4	0.7–2.1	0.4–0.6	0.5–1.5	0.2–0.6	0.9–1.5
3-methyl-1-butanol (Isoamyl alcohol)	721		11.0–24.0	22.9–28.6	19.8–45.0	10.8–71.8	11.5–37.8	19.9–27.5	19.3–44.2	39.0–43.1	17.6–37.8	17.8–28.9	9.9–40.4	20.7–51.6	21.7–41.3	10.1–20.9	23.8–44.1	28.4–50.8	5.1–6.8	5.6–11.4	5.6–22.8	17.6–32.1	12.4–19.1	16.8–42.0	6.8–19.1	15.6–25.9
2-methyl-1-butanol (Amyl alcohol)	722		0.0–3.0	6.5–10.7	5.1–9.0	4.3–20.2	2.7–6.9	5.5–8.9	2.8–7.3	6.0–9.4	5.4–12.5	4.9–8.3	2.4–7.1	4.4–13.4	4.4–10.6	1.9–5.7	5.1–7.5	5.9–11.5	1.8–2.4	2.3–4.9	1.3–3.7	4.0–8.4	2.7–3.9	4.7–12.3	1.9–3.6	3.5–7.0
2,3-butanediol	756		0.1–0.3	0.0–0.1	0.0–0.2	0.0–0.2	<0.1–0.1	0.0–0.5	0.0–0.1	0.0–0.4	0.0–0.1	Nd	0.0–0.3	Nd	0.0–0.9	Nd	0.0–0.1	Nd	<0.1–0.1	Nd	<0.1–0.2	Nd	0.0–0.1	Nd	<0.1	0.0–0.4

2-phenylethanol	1133		1.1–11.4	0.5–7.1	7.4–13.4	0.2–2.0	3.7–8.3	2.2–3.5	1.3–3.0	3.3–15.4	0.6–8.0	0.8–2.8	2.8–10.2	2.2–4.0	0.0–6.2	0.5–2.6	1.5–7.1	0.9–3.7	1.4–1.8	0.0–0.6	1.5–2.6	0.0–1.4	1.3–3.5	0.0–0.2	0.7–2.7	0.0–2.9
3,7–dimethyl–6–octen–1–ol (Citronellol)	1235		0.1–0.4	0.1–0.4	0.1–0.2	Nd	0.1	0.0–0.2	0.0–0.1	0.2–0.4	0.0–0.2	Nd	<0.1–0.2	0.0–0.4	0.0–0.1	0.0–0.2	0.0–0.1	0.0–0.2	0.4–0.7	0.0–0.1	0.2–0.4	0.0–0.2	0.2–0.3	0.0–0.1	0.2–0.4	0.0–0.2
2,4-bis(1,1-dimethylethyl)-phenol	1516		0.3–2.2	1.2–3.1	0.5–1.0	1.6–3.1	0.3–0.7	0.7–2.0	0.2–0.3	1.2–5.7	0.0–0.6	0.6–4.2	0.0–0.7	1.5–7.3	0.0–0.2	1.6–10.0	0.0–0.6	0.9–2.1	0.3–1.6	0.3–17.3	0.2–0.5	1.4–4.5	0.5–0.7	1.7–17.9	0.3–0.8	0.9–2.3
3,7,11-trimethyl-1,6,10-dodecatrien-3-ol (Nerolidol)	1565		0.0–0.4	0.1–0.4	0.1–0.2	Nd	<0.1–0.1	Nd	0.0–0.1	<0.1–0.5	0.0–0.1	0.0–0.1	0.0–0.2	0.0–11.3	Nd	0.0–0.1	0.0–0.1	Nd	0.1	Nd	0.1	Nd	0.1	Nd	<0.1–0.2	0.0–0.1
3,7,11-trimethyl-2,6,10-dodecatrien-1-ol	1726		0.0–0.2	0.0–0.3	<0.1–0.1	Nd	Nd	0.0–0.1	0.0–<0.1	0.0–0.3	Nd	Nd	0.0–0.2	0.0–0.2	Nd	0.0–0.3	Nd	Nd	Nd	Nd	0.1	0.0–0.1	<0.1–0.1	Nd	0.0–0.1	0.0–0.1
Total alcohols			**16.2–39.4**	**41.6–45.2**	**40.5–60.6**	**18.9–102.6**	**18.7–54.3**	**30.4–42.7**	**27.1–52.0**	**51.6–76.9**	**28.8–52.4**	**25.9–43.6**	**24.4–51.1**	**32.2–83.4**	**30.3–53.9**	**17.6–37.3**	**34.2–55.9**	**40.5–68.0**	**9.7–13.2**	**8.9–33.6**	**10.7–28.7**	**25.4–47.1**	**18.6–27.8**	**23.8–73.9**	**10.0–26.0**	**25.9–35.3**
State of the cells		9.91**																								
Nature of kefir culture		4.91**																								
Fermentation temperature		14.54**																								
All interactions		0.84																								
Carbonyl compounds																										

Benzaldehyde	947		0.0–0.2	0.0–0.4	0.2–0.3	Nd	0.0–0.2	<0.1–0.1	0.0–<0.1	<0.1–0.1	Nd	Nd	0.0–0.1	0.0–0.2	Nd	0.0–<0.1	Nd	Nd	Nd	Nd	Nd	Nd	Nd	Nd	Nd	Nd
Nonanal	1128		0.0–0.1	0.0–0.1	0.0–0.2	Nd	0.0–0.1	0.0–0.1	Nd	0.1–1.0	0.0–0.2	Nd	Nd	Nd	Nd	Nd	0.0–0.2	Nd	0.1	0.0–0.1	Nd	Nd	Nd	Nd	0.0–0.2	0.0–0.1
4-propyl-benzaldehyde	1275		Nd	0.0–0.1	Nd	Nd	Nd	0.0–0.1	Nd	0.1–0.3	Nd	Nd	Nd	Nd	Nd	Nd	Nd	Nd	Nd	Nd	Nd	Nd	Nd	Nd	Nd	0.0–0.2
(E)-1-(2,6,6-trimethyl-1,3-cyclohexadien-1-yl) -2-Buten-1-one (β-damascenone)	1384		0.0–0.2	0.0–0.1	0.1–0.2	Nd	Nd	Nd	0.0–0.1	<0.1–0.2	Nd	Nd	0.0–0.2	0.0–<0.1	Nd	Nd	0.0–0.3	0.0–0.5	<0.1–0.1	Nd	<0.1–0.1	Nd	<0.1–0.2	Nd	0.0–<0.1	0.0–0.6
Total carbonyl compounds			**0.0–0.4**	**0.0–0.7**	**0.3–0.7**	**Nd**	**0.0–0.3**	**0.1–0.2**	**0.0–0.1**	**0.5–1.1**	**0.0–0.2**	**Nd**	**0.0–0.3**	**0.0–0.2**	**Nd**	**Nd**	**0.0–0.5**	**0.0–0.5**	**0.1–0.2**	**0.0–0.1**	**<0.1–0.1**	**Nd**	**<0.1–0.2**	**Nd**	**<0.1–0.2**	**0.0–0.7**
State of the cells		1.03																								
Nature of kefir culture		8.42**																								
Fermentation temperature		15.69**																								
All interactions		6.50**																								
Miscellaneous compounds																										
2-fluoro-1-propene	<700		0.0–0.3	0.3–1.9	0.0–0.4	0.2–3.1	0.0–0.3	0.2–1.0	0.0–0.4	0.5–0.9	0.2–2.0	0.3–1.0	0.0–0.8	0.2–1.7	0.0–1.4	0.1–0.8	0.3–1.2	0.5–1.9	0.0–0.2	0.0–0.1	0.0–0.6	0.5–4.1	0.0–0.4	0.5–2.7	0.2–0.4	0.6–2.7
2,5-dimethyl-furan	704		0.1	0.0–<0.1	0.0–0.1	Nd	0.0–<0.1	0.0–0.2	0.0–<0.1	<0.1	0.0–0.1	0.0–0.1	0.0–0.1	0.0–0.1	0.0–0.1	0.0–0.1	0.0–0.1	Nd	<0.1–0.1	Nd	<0.1–0.1	Nd	0.0–0.1	0.0–0.2	0.0–<0.1	Nd

1,1-diethoxy-ethane (Acetal)	716	0.8–2.9	0.5–0.9	0.0–0.3	0.7–1.2	0.1–0.9	0.3–1.0	Nd	0.3–0.7	1.1–4.9	0.1–0.9	0.0–1.3	0.1–2.0	0.0–1.2	0.1–1.1	0.2–1.0	0.2–0.6	1.0–2.8	0.4–1.8	0.0–0.6	0.6–6.2	0.3–0.7	1.0–1.1	0.2–0.7	0.5–1.3
Toluene	735	0.0–0.2	0.0–0.1	0.0–0.2	Nd	0.0–0.2	0.0–0.1	Nd	0.0–0.2	0.0–0.1	Nd	0.0–0.1	Nd	0.0–0.2	Nd	0.0–0.2	Nd	0.1–0.2	Nd	0.0–0.1	0.0–0.1	Nd	Nd	0.1–0.2	Nd
2,4-dimethyl-heptane	815	Nd	0.0–0.1	Nd	Nd	Nd	Nd	Nd	0.0–0.1	Nd	0.0–0.2	Nd	0.0–0.2	Nd	0.0–0.2	Nd	0.0–0.3	0.0–<0.1	0.0–0.1	Nd	0.0–0.1	Nd	Nd	<0.1	0.0–<0.1
1,3,5-trimethyl-benzene (Mesitylene)	956	0.3–0.6	0.2–0.3	0.4–0.7	0.0–0.1	0.4–0.5	0.2–0.3	0.1–0.4	0.3–0.8	0.4–0.5	0.0–0.4	0.3–0.6	0.0–0.6	0.0–0.4	0.0–0.4	0.0–0.5	0.0–0.4	0.4–0.6	0.0–0.2	0.3–0.5	0.0–0.4	0.3–0.4	0.0–0.1	0.3–0.4	0.0–0.3
Decane	999	Nd	0.0–<0.1	Nd	Nd	Nd	Nd	Nd	0.0–<0.1	Nd	Nd	Nd	0.0–0.2	Nd	0.0–0.2	Nd	0.0–0.4	0.0–0.1	Nd	0.0–0.1	Nd	Nd	Nd	0.0–0.1	0.0–<0.1
4-methyl-decane	1023	<0.1–0.1	0.0–0.1	0.0–0.1	Nd	0.0–0.1	0.0–0.1	Nd	0.1	Nd	0.0–<0.1	0.0–<0.1	0.0–0.3	Nd	0.0–0.4	Nd	0.0–0.1	0.0–0.1	Nd	0.0–0.1	Nd	0.0–0.1	Nd	0.0–<0.1	0.1–0.5
4,7-dimethyl-undecane	1067	0.0–0.2	0.0–0.2	0.0–0.2	0.0–<0.1	0.2	0.0–0.1	0.0–0.1	0.1–0.2	0.0–0.2	0.0–0.3	0.0–0.2	Nd	0.0–0.1	Nd	0.0–0.2	Nd	0.1–0.3	0.0–0.1	0.0–0.2	0.0–0.2	0.0–0.1	Nd	0.0–0.1	0.0–<0.1
2,6-dimethyl-undecane	1216	0.1–0.3	0.0–0.1	0.0–0.3	Nd	0.1–0.2	0.0–0.1	Nd	<0.1–0.4	0.0–0.1	0.0–0.4	0.1–0.3	0.0–0.5	0.0–0.1	0.0–0.2	Nd	0.0–0.3	0.1–0.2	0.0–0.1	0.1–0.2	0.0–0.1	0.0–0.2	Nd	0.1–0.2	0.0–0.1
4,8-dimethyl-undecane	1226	<0.1–0.1	0.0–0.1	0.1–0.2	Nd	0.1	0.0–0.1	0.0–0.1	0.0–0.2	0.0–0.1	Nd	Nd	Nd	0.0–0.1	Nd	0.0–0.1	Nd	0.1	Nd	0.1	0.0–0.1	0.1–0.2	Nd	<0.1–0.1	0.0–0.1

1,3-bis(1,1-dimethylethyl) -benzene (m-Di-tert-butylbenzene)	1258		0.8–1.9	0.9–1.3	1.0–2.1	0.3–0.4	1.0–1.4	0.3–1.1	0.4–1.3	1.1–3.3	0.8–1.5	1.6–5.8	0.6–2.2	3.0–6.6	0.3–1.4	1.2–3.6	0.1–1.9	1.5–3.5	1.3–2.3	0.7–7.4	1.2–1.7	1.2–1.9	1.2–1.5	0.7–3.1	1.0–1.9	0.3–1.2
2,4-dimethyl-undecane	1272		0.0–0.3	0.0–0.2	<0.1–0.5	Nd	0.0–0.2	0.0–0.2	0.0–0.1	0.1–0.6	0.0–0.2	Nd	Nd	Nd	Nd	Nd	Nd	Nd	0.0–0.2	Nd	0.0–0.1	Nd	0.0–0.2	Nd	0.0–0.2	0.0–0.1
2,6,10-trimethyl-dodecane	1303		<0.1–0.2	0.0–0.1	0.0–0.2	Nd	0.1	0.0–<0.1	Nd	Nd	0.0–0.2	Nd	0.0–0.2	Nd	0.0–0.1	Nd	0.0–0.3	Nd	0.1–0.4	0.0–1.0	0.0–0.2	0.0–0.1	0.0–0.1	0.0–0.1	Nd	Nd
Tetradecane	1399		0.0–0.3	0.0–0.3	0.3–0.4	Nd	0.2–0.3	0.0–0.1	Nd	0.1–0.8	Nd	0.0–0.5	0.0–0.3	0.0–0.2	0.0–0.2	0.0–0.2	0.0–0.3	0.0–0.2	0.1–0.3	0.0–1.6	0.2–0.3	Nd	0.2–0.3	0.0–0.3	0.1–0.3	0.0–0.2
Pentadecane	1503		0.1–0.3	0.1–0.2	0.2–0.4	Nd	0.1–0.2	<0.1–0.1	0.0–0.1	0.2–0.3	0.0–0.1	0.0–0.1	0.1–0.3	0.0–0.6	0.0–0.1	0.0–0.1	0.0–0.2	0.0–0.2	0.1–0.2	0.1–1.1	0.2–0.3	0.2–0.4	0.2–0.3	0.0–0.2	0.1–0.2	0.0–0.2
Heptadecane	1702		0.0–0.1	0.0–0.1	0.0–0.1	Nd	0.0–<0.1	Nd	Nd	Nd	Nd	Nd	0.0–0.2	Nd	0.0–0.1	Nd	Nd	Nd	0.0–<0.1	Nd	<0.1–0.1	Nd	<0.1–0.1	0.0–1.2	0.0–0.1	0.0–<0.1
Total miscellaneous compounds			**4.3–7.2**	**3.4–4.0**	**2.4–5.5**	**1.3–4.7**	**3.0–3.9**	**1.9–3.0**	**0.6–2.1**	**3.2–8.5**	**3.8–8.4**	**3.3–8.1**	**3.2–4.6**	**4.7–10.1**	**2.1–3.6**	**1.8–6.2**	**2.3–4.3**	**3.3–5.7**	**3.8–7.7**	**2.0–12.3**	**3.6–3.8**	**3.6–12.6**	**3.3–4.1**	**2.6–8.0**	**2.2–4.1**	**3.7–4.1**
State of the cells		4.19*																								
Nature of kefir culture		2.82*																								
Fermentation temperature		2.60																								
All interactions		1.34																								
Total volatiles			**75.0–156.0**	**77.5–80.6**	**114.0–147.1**	**40.1–132.0**	**71.1–132.9**	**70.7–117.1**	**88.1–220.7**	**137.9–224.4**	**130.2–221.6**	**60.8–129.6**	**116.3–254.4**	**105.2–337.8**	**91.5–188.3**	**122.7–242.3**	**97.0–368.1**	**127.4–239.2**	**82.6–90.1**	**27.8–124.7**	**90.3–101.9**	**120.6–274.9**	**126.7–164.3**	**96.1–365.3**	**71.9–171.1**	**64.1–126.8**

State of the cells	0.55
Nature of kefir culture	4.47**
Fermentation temperature	8.72**
All interactions	1.19

W: Wet cells, Fd: Freeze-dried cells; Nd: Not detected; *$p < 0.05$, **$p < 0.01$.
